# In evolution’s unending race: ancestral STING sensors in *Salmo salar* mediate intracellular bacterial detection and programmed cell death through evolutionarily conserved pathways

**DOI:** 10.3389/fimmu.2025.1570871

**Published:** 2025-06-18

**Authors:** Alejandro J. Yañez, Jorge F. Beltrán, Claudia A. Barrientos, Genaro Soto-Rauch, Marcelo Aguilar, Adolfo Isla, Sandra N. Flores-Martin, Francisco T. Yañez, Yassef Yuivar, Adriana Ojeda, Felipe Almendras, Patricio Bustos, Marcos Mancilla

**Affiliations:** ^1^ Departamento de Investigación y Desarrollo, Greenvolution SpA, Puerto Varas, Región de Los Lagos, Chile; ^2^ Interdisciplinary Center for Aquaculture Research (INCAR), Concepción, Chile; ^3^ Laboratorio de Diagnóstico y Terapia, Vicerrectoría de Investigación, Desarrollo y Creación Artística (VIDCA), Universidad Austral de Chile, Valdivia, Chile; ^4^ KeyBio Solution, Valdivia, Región de los Rios, Chile; ^5^ Department of Chemical Engineering, Universidad de la Frontera, Temuco, Chile; ^6^ Departamento de Ciencias Básicas, Facultad de Ciencias, Universidad Santo Tomas, Valdivia, Chile; ^7^ Laboratorio de Diagnóstico y Biotecnología, ADL Diagnostic Chile, Puerto Montt, Chile

**Keywords:** STING gene, dynamics activation gene expression, structural functions, innate immune response, Atlantic salmon-pathogen interaction, evolutionary perspectives

## Abstract

**Introduction:**

“In evolution’s unending race, survival demands continuous adaptation— to stop is to fall behind.” The Stimulator of Interferon Genes (STING) pathway embodies this principle, acting as a conserved master regulator of cytosolic DNA sensing from *Drosophila* to salmon and humans. Although extensively characterized in mammals, its structural features and regulatory roles during intracellular bacterial infection in teleosts remain poorly defined.

**Methods:**

We structurally characterized the ancestral STING ortholog from Atlantic salmon (*Salmo salar*) using AlphaFold-guided modeling to identify conserved motifs, including the cyclic dinucleotide (CDN)-binding cleft and phosphorylation regulatory sites. Molecular docking simulations were performed to evaluate the interaction of a validated human STING agonist with salmonid STING. Transcriptomic analyses were conducted in immune tissues and SHK-1 macrophage-like cells infected with *Piscirickettsia salmonis* to assess gene expression dynamics.

**Results:**

Our models confirmed evolutionary conservation of key STING structural domains. Docking revealed a strong binding affinity between the human agonist and salmonid STING, supporting translational potential. Transcriptomics showed high *sting1* expression in immune tissues, rapidly upregulated after infection. In SHK-1 cells, STING1, IFN-α, TNF-α, and IL-1β peaked at 4 hours post-infection (hpi), but this inflammatory burst collapsed by 5 days post-infection (dpi), despite persistent *sting1* transcription, indicating functional uncoupling due to immune evasion. *In vivo*, prolonged DDX41–STING activation was associated with reduced pyroptosis, necroptosis, and inflammatory signaling, reflecting bacterial suppression mechanisms.

**Discussion:**

This study positions *S. salar* as a high-resolution model for STING biology and introduces the Evolutionary Molecular Immunity Race (EMIR) framework, where STING orchestrates immune fate across hundreds of millions of years of vertebrate evolution, and over the last ~80 million years within the salmonid lineage.

## 1 Introduction

“In evolution’s unending race, survival is earned only through continuous adaptation—to stop is to fall behind.” This principle defines the molecular architecture of innate immunity, emphasizing that survival is not a passive state but an active genomic adaptation. In teleost fish, the innate immune system constitutes the first line of defense against microbial threats, orchestrated by intricate networks of pattern recognition receptors (PRRs) and their associated signaling pathways (1). These receptors detect pathogen-associated molecular patterns (PAMPs), initiating immune responses such as inflammation, cytokine production, and cell death pathways to eliminate pathogens and maintain cellular homeostasis (2). Among these, cytosolic DNA sensors have emerged as a pivotal evolutionary innovation, enabling organisms to detect intracellular pathogens and mount rapid immune defenses (3, 4).

A key component of this sensing network is the cyclic GMP–AMP synthase–Stimulator of Interferon Genes (cGAS–STING) signaling axis, which is highly conserved from invertebrates to vertebrates (5). Upon recognition of cytosolic double-stranded DNA (dsDNA), cGAS synthesizes cyclic GMP–AMP (cGAMP), which binds to and activates STING. Activated STING then recruits TBK1, which phosphorylates the transcription factors IRF3 and IRF7, ultimately triggering a robust type I interferon and pro-inflammatory response (6–8). While extensively characterized in mammals, the ancestral roles of STING precedes the vertebrate interferon system. In *Drosophila melanogaster*, STING homologs regulate essential cellular processes such as lipid metabolism, autophagy, and innate antiviral responses independently of interferon signaling, suggesting that early evolutionary functions were rooted in general cellular stress responses and immune balance (9–11).

In teleosts, STING was first identified as crucian carp (*Carassius carassius*), where it exhibited conserved antiviral activity via interferon signaling pathways (12). Subsequent studies in grouper and other fish species confirmed these functions, reinforcing STING’s evolutionary conservation across vertebrates (13, 14). Notably, several teleost viruses—including Singapore Grouper Iridovirus (SGIV) and Grass Carp Reovirus (GCRV)—have evolved sophisticated mechanisms to inhibit STING activity, highlighting the intense evolutionary arms race between host immunity and pathogen evasion (15, 16).

Against this evolutionary backdrop, the present study investigates the molecular evolution and functional conservation of the STING gene, tracing its trajectory from ancestral invertebrates to specialized roles in Atlantic salmon (*Salmo salar*) (9, 10, 12–14). Utilizing advanced structural modeling via AlphaFold (17, 18), we demonstrate substantial conservation of critical structural motifs in salmonid STING—such as cyclic dinucleotide-binding domains (7, 19), palmitoylation sites essential for membrane localization (20), and autophagy-related motifs that contribute to immune homeostasis (21, 22). These findings support the notion of structural and functional orthology of STING across vertebrate lineages (23, 24), positioning *S. salar* as a valuable model for dissecting STING-mediated immunity with direct implications for disease control in aquaculture (25, 26).

Throughout vertebrate evolution, STING has expanded its functional repertoire beyond interferon induction to include the orchestration of diverse immune processes such as apoptosis, necroptosis, pyroptosis, and PANoptosis (27, 28). This functional plasticity underscores its role as a master regulator of immune responses, enabling context-dependent decisions in response to pathogen burden and cellular stress. In turn, pathogens have evolved precise mechanisms—including proteases, ubiquitin ligases, and regulatory non-coding RNAs—to subvert STING signaling, exemplifying the ongoing co-evolutionary conflict between host defense and microbial evasion (29–33).

In salmonids, intracellular pathogens such as *Piscirickettsia salmonis* exploit the macrophage intracellular niche and are hypothesized to employ convergent immune evasion strategies analogous to those observed in mammals and other teleosts. Dissecting the regulation of STING during bacterial infections in salmon not only provides insight into innate immune evolution but also informs the development of novel strategies to enhance pathogen resistance in aquaculture (25, 34).

To advance this evolutionary perspective, we introduce the Evolutionary Molecular Immunity Race (EMIR) a conceptual framework that redefines STING as more than a mere pathogen sensor. EMIR proposes that STING acts as a multifaceted immune algorithm shaped by six evolutionary regulatory layers: immune signaling, autophagy, programmed cell death, immune modulation, proteolysis, and epigenetic silencing. Each layer represents an adaptive solution to evolutionary pressures, enabling STING to coordinate precise immune responses across diverse biological contexts. EMIR therefore conceptualizes STING as an evolutionary integrator that balances immune activation, pathogen tolerance, and homeostatic control. This framework offers transformative implications for both basic immunology and translational applications in disease management.

To substantiate our model, we employed a rigorous suite of methodologies: orthology assessment using OMA datasets (35), sequence homology searches with BLAST (36), transcript quantification with Kallisto (37), and *de novo* transcriptome assembly (38). Structural alignments were conducted using fold-recognition algorithms (39), while insights into subcellular trafficking were informed by recent advances in STING biology (40). These integrative approaches collectively reinforce the depth and precision of our evolutionary and functional exploration within the EMIR framework.

By reconstructing STING’s evolutionary pathway from *Drosophila* to *S. salar*, we reveal an underlying continuity in structural design alongside dynamic functional adaptation—hallmarks of immunological resilience. This evolutionary analysis not only refines our understanding of innate immune logic but also holds practical value in both human medicine and aquaculture. Through EMIR, we offer a comprehensive lens for decoding the regulatory architecture of STING and propose a robust platform for future immunological innovation.

## 2 Materials and methods

### 2.1 Identification and characterization of the STING gene in *Salmo salar*

The STING homolog gene, including its transcript variants and isoforms in *Salmo salar*, was identified by searching publicly available databases, including the National Center for Biotechnology Information (NCBI), ENSEMBL, GenBank and SalmonBase (https://salmobase.org/). This search was guided by previously reported information on the *Homo sapiens* STING gene, including its genomic location, accession number, and assembly information (GRCh38.p14; NC_000005.10). Additionally, mRNA variants and their corresponding protein isoforms (NP_938023.1, NP_001288667.1, NP_001354187.1) were retrieved from the NCBI database.

The *S. salar sting* gene was identified using the *H. sapiens sting1* homolog as a reference. BLASTn and BLASTp (36) were employed to search against the *S. salar* genome assembly and CDS collection available in ENSEMBL (Ssal_v3.1). The retrieved sequences, including the gene, mRNA, and coding sequence (CDS), were visualized using the pyGenomeViz package (41). Additionally, protein and variant analyses were performed using *H. sapiens* isoform 1, identified as the closest match in BLAST searches. Finally, the gene was validated in the transcriptome assembly (GGAQ00000000.1) (38).

### 2.2 Evolutionary and phylogenetic analysis of *sting1*

Complete mRNA sequences from 39 representative vertebrate species (**Supplementary Table 1**) were retrieved from NCBI (accessed January 20, 2024). Sequences were aligned using Clustal Omega (42) with the Gonnet substitution matrix. Alignments were manually curated. Phylogenetic trees were constructed using the Maximum Likelihood method in MEGA 11 (43) with 10,000 bootstrap replicates and visualized using iTOL (44).

To determine the evolutionary relationship of *S. salar sting1* with homologs from other species, we retrieved complete mRNA sequences from 39 representative vertebrates available in the NCBI database (accessed on January 20, 2024) (**Supplementary Table 1**). These species and sequence were selected based on three primary criteria: (i) Phylogenetic diversity, ensuring the inclusion of major vertebrate lineages—teleost fish (particularly salmonids), amphibians, reptiles, birds, and mammals—(ii) Availability of high-quality complete mRNA sequences to ensure reliable comparisons and (iii) Best results hit within BLAST search using both *H. sapiens* and *S. salar*.

Emphasis was placed on salmonids such as *S. salar*, *S. trutta*, *O. mykiss* and *O. nerka* to reflect the study’s aquaculture relevance, while broader vertebrate representation (*D. rerio*, *X. laevis*, *H. sapiens*) enabled the assessment of deep evolutionary conservation. Retrieved sequences were aligned using Clustal Omega (42) with default parameters, including gap opening penalty, gap extension penalty, and substitution matrix (default: Gonnet). Alignments were manually curated to eliminate poorly aligned or ambiguous regions, ensuring accurate positional homology. Only high-confidence alignment blocks were retained for phylogenetic analysis. A phylogenetic tree was constructed using the Maximum Likelihood (ML) method in MEGA 11 (43). Bootstrap analysis was performed with 10,000 replicates to assess branch support. The resulting tree was exported in Newick format and visualized using iTOL (44).

### 2.3 Structural and functional analysis of STING1 in *S. salar*

Three-dimensional (3D) structures of STING from *S. salar* (XP_014068485.1) and *H. sapiens* (NP_938023.1) were predicted using AlphaFold2 in no-template mode under default settings (17, 45). For each protein, five models were generated, and the highest-confidence structure, based on predicted Local Distance Difference Test (pLDDT) scores, was selected for subsequent analyses. Model quality was rigorously evaluated using ERRAT (46) to assess non-bonded atomic interactions, VERIFY3D (39, 47) to examine compatibility between 3D structures and 1D sequences, and PROCHECK (48) to assess stereochemical geometry, including Ramachandran plot distributions. Final structural models were visualized and analyzed using PyMOL v3.0.3 (49).

### 2.4 Comparison of STING-TM and STING-C domains across species

The functional analysis focused on two critical regions of STING: (i) The Transmembrane (TM) domain, which anchors STING to the endoplasmic reticulum membrane—a prerequisite for signal transduction upon activation. (ii) The C-terminal (C) domain, which binds cyclic dinucleotides (CDNs) such as cGAMP and mediates interactions with downstream adaptors including TBK1 and IRF3, triggering immune gene activation. Understanding the conservation of these domains is essential for evaluating functional retention across evolutionary lineages.

The predicted STING coding sequences of *S. salar* and *H. sapiens* were translated into amino acid sequences using EMBOSS Transeq (50). Protein domain coordinates for STING-TM and STING-C were retrieved from the OMA browser (35) for seven representative species, including: three fish *S. salar*: XP_014068485.1, *Xiphophorus maculatus*: XP_014328830.1, *D. rerio*: NP_001265766.1), one amphibian (*Xenopus laevis*: XP_041443985.1), one bird (*Meleagris gallopavo*: XP_010717095.1), and two mammals (*Mus musculus*: XP_017173483.1, *H. sapiens*: AVQ94753.1). Multiple sequence alignments were conducted with ClustalW with default parameters, including gap opening penalty, gap extension penalty, and substitution matrix (default: BLOSUM). The resulting alignments were visualized using Jalview (51), and analyzed based on conservation, sequence quality, consensus, and occupancy scores.

To further assess sequence similarity, domain-specific alignments were extracted and analyzed using BLASTp, comparing STING-TM and STING-C domains across species. The sequence similarity percentages were determined using the S. salar domain coordinates as a reference.

### 2.5 Molecular docking analysis

#### 2.5.1 Docking of STING (*S. salar* and *H. sapiens*) with HB3089

Docking analyses were conducted with c-di-GMP extracted from PDB ID: 4EMT. Both *H. sapiens* STING (8GT6) and *S. salar* STING models were used as receptors, maintaining docking parameters optimized for each structure. Molecular docking was performed using AutoDock Vina (52) via PyRx (53). The *S. salar* STING model generated by AlphaFold3 (17) served as the receptor, and HB3089, a human STING agonist co-crystallized with Homo sapiens STING (PDB ID: 8GT6), was used as the ligand. The ligand was extracted from the 8GT6 structure, energy-minimized using the UFF force field, and docked into the native binding pocket. Docking parameters were set with a grid box centered at x = −19.24, y = −7.21, z = 2.71, and dimensions of 25.0 × 26.29 × 25.0 Å³, with an exhaustiveness value of 8. Docking results were visualized and analyzed using PyMOL v3.0.3 (49).

#### 2.5.2 Docking of STING (*H. sapiens* and *S. salar*) with c-di-GMP

Similarly, c-di-AMP (PubChem CID: 11158091) was docked against both STING models, with interaction parameters tailored to native binding sites. All docking results were visualized and analyzed with PyMOL v3.0.3. Docking analyses were performed using AutoDock Vina (52) via PyRx (53). The *H. sapiens* STING crystal structure (PDB ID: 8GT6) served as the receptor, with c-di-GMP extracted from PDB ID: 4EMT (54) as the ligand. After energy minimization using the UFF force field, the ligand was docked at its native binding site. Interaction box parameters were center_x = 51.23, center_y = 18.61, center_z = 67.95; size_x = 25.0, size_y = 25.0, size_z = 25.0, size_z = 25.0, with exhaustiveness = 8. A second docking used the *S. salar* STING model generated by AlphaFold 3 (17) as the receptor, with c-di-GMP extracted from PDB ID: 4EMT. The interaction box parameters were center_x = -11.21, center_y = -4.17, center_z = 5.07; size_x = 34.96, size_y = 42.70, size_z = 37.81, with exhaustiveness = 8. Both results were visualized using PyMOL v3.0.3 (17).

#### 2.5.3 Docking of STING (*H. sapiens* and *S. salar*) with c-di-AMP

Similarly, c-di-AMP (PubChem CID: 11158091) was docked against both STING models, with interaction parameters tailored to native binding sites. All docking results were visualized and analyzed with PyMOL v3.0.3. Docking was performed using AutoDock Vina (52) via PyRx (53). The *H. sapiens* STING crystal structure (PDB ID: 8GT6) served as the receptor, with c-di-AMP obtained from PubChem (55) (CID: 11158091) as the ligand. The ligand was energy-minimized using the UFF force field and docked at the native binding site. Interaction box parameters were center_x = 134.23, center_y = 135.02, center_z = 86.24; size_x = 25.0, size_y = 36.98, size_z = 25.0, with exhaustiveness = 8. A second docking used the *S. salar* STING model generated by AlphaFold 3 (17) as the receptor and the same ligand. The interaction box parameters were center_x = -25.25, center_y = -15.27, center_z = 8.92; size_x = 28.30, size_y = 24.76, size_z = 32.82, with exhaustiveness = 8. Results were visualized using PyMOL v3.0.3 (49).

### 2.6 RNA-seq transcript quantification

Publicly available RNA-Seq dataset from the *S. salar* transcriptome were retrieved from public databases using the SRA Toolkit (https://trace.ncbi.nlm.nih.gov/Traces/sra/sra.cgi?view=software). The dataset included control conditions from various experiments and tissues: liver (SRR18473589, SRR18473590, SRR18473591) (56), intestine (SRR22335968, SRR22335970, SRR22335972) (57), gill (SRR16991289, SRR16991290, SRR16991291) (58), spleen (SRR17487648, SRR17487649, SRR17487650) (59), kidney (SRR12187260, SRR12187261, SRR12187262) (60), and brain/ovary (SRR7139945–SRR7139963) (61). **Preprocessing and Mapping**. The paired-end raw sequencing reads were assessed for quality control using FastQC (62). High-quality reads were then mapped to the *S. salar* transcriptome (Ssal_v3.1) using Kallisto v0.46.1 (37). **Normalization and Statistical Analysis**. Gene expression counts were normalized using the variance stabilizing transformation (VST) in DESeq2 (63). Statistical comparisons of *sting1* expression across tissues were conducted using one-way ANOVA, followed by Tukey’s *post-hoc* test (64, 65) to assess pairwise differences.

### 2.7 Primer design and qPCR analysis

The primer sets for *sting1* in *S. salar* were designed using Primer-BLAST (66) based on sequences retrieved from NCBI (Accession number: NC_059450.1). The primer sets for *irf3*, *ifn*-γ, *elf*-1α, *ddx41*, *il*-1β, and *tnf*-α in *S. salar*, as well as the primer set for *P. salmonis* detection (Psal_detec), were previously published. The sequences and corresponding references are detailed in **Table 1**.

**Table 1:** Primer sequences used for quantitative real-time PCR (qPCR) analyses.

Gene name	Forward sequence	Reverse sequence	Accession number or reference
*elf-*1α	CCCCTCCAGGACGTTTACAAA	CACACGGCCCACAGGTACA	67
*sting1*	CCCGTTTGCCCAATTTGAAGT	AAGAGGCTTTTCGCCGTCAT	This work
*irf*3	GCAGAGGGGATCTCAACCAC	GTGCCACATTGGAACGGTTG	68
*ddx41*	GCCAGCTTGGACGTCATTCAG	CTGGTCTTTTCCTCCGTGGAT	68
*inf-γ*	CGTGTATCGGAGTATCTTCAACCA	CTCCTGAACCTTCCCCTTGAC	68
*il-1β*	CAAGCTGCCTCAGGGTCT	CGCCACCCTTTAACCTCTCC	68
*tnf-α*	CGTGGTGTCAGCATGGAAGA	AGTATCTCCAGTTGAGGCTCCATT	68
Psal_detec	GCTGTGCCCAGAACTTTAG	GACCACTRCCTTTACCAAAC	69

The table includes primers for the reference gene *elf-1α*, sting pathway-associated genes (*sting1*, *irf3*, *ddx41*, *inf-γ*, *il-1β*, and *tnf-α*), and a primer set for the detection of *P. salmonis* (*Psal_detec*). Primers were either designed in this study (*sting1*) or obtained from previously published sources, as indicated. Primer specificity and amplification efficiency were validated prior to use.

RNA Extraction and cDNA Synthesis. Total RNA was extracted from fish tissues and cell cultures using TRIzol reagent (Life Technologies, Carlsbad, CA, USA) following the manufacturer’s protocol. RNA concentration and purity were assessed using a Qubit 3 Fluorometer (Life Technologies, Carlsbad, California, US) with the Qubit RNA BR Assay Kit (Life Technologies, Carlsbad, CA, USA). For cDNA synthesis, 2 µg of total RNA was reverse transcribed using M-MLV Reverse Transcriptase (Promega, Madison, WI, USA) and oligo(dT) primers (Promega, Madison, WI, USA), following the manufacturer’s instructions.

qPCR Conditions and Data Analysis. Quantitative real-time PCR (qPCR) was performed using an Mx3005P system (Stratagene, La Jolla, CA, USA) with Brilliant II SYBR Green QPCR Master Mix (Agilent Technologies, Santa Clara, CA, USA). The thermal cycling conditions were as follows: Initial denaturation: 10 min at 95°C. Amplification (40 cycles): 15 s at 95°C, 1 min at 60°C. Final steps: 15 s at 72°C, 30 s at 58°C, 15 s at 95°C.

To ensure specificity, melting curve analysis was performed at the end of each run. mRNA expression levels were normalized to the housekeeping gene *elf*-1α using the comparative Ct (2^−ΔΔCt^) method (70). All reactions were performed in triplicate, with at least three independent biological replicates per condition.

### 2.8 Cell line culture and bacterial infection assays

The SHK-1 cell line, derived from *S. salar* macrophages, was used for infection kinetics assays (25, 71). Cells were maintained at 18°C in Leibovitz’s L-15 medium (Gibco, Carlsbad, CA, USA) supplemented with 10% (v/v) fetal bovine serum (FBS, HyClone, South Logan, UT, USA) and without antibiotics. Cultures were grown to 90% confluency in 25 cm² flasks at 18°C. Twenty-four hours prior to infection, cells were incubated in Leibovitz’s L-15 medium containing 2% (v/v) FBS at 18°C.

Bacterial Culture and Identification. For *in-vitro* assays, *P. salmonis* was cultured in Austral-SRS broth at 18°C with shaking at 180 rpm for 5 days (72, 73). Bacterial morphology and identity were confirmed using Gram staining, PCR (69), Loop-mediated isothermal amplification (qPCR and LAMP) assay (74) and Immunofluorescence antibody testing using the Salmonid Rickettsial Septicemia (SRS) Immunofluorescence KIT (Ango, San Ramon, CA, USA), following the manufacturer’s instructions.

Infection Assay. SHK-1 cells were inoculated with *P. salmonis* genogroups (75) at a multiplicity of infection (MOI) of 10 CFU/cell in 3 mL of Leibovitz’s L-15 medium (68). After 2 hours of incubation at 18°C, cells were washed with sterile 1× Phosphate-Buffered Saline (PBS) to remove non-internalized bacteria. Fresh Leibovitz’s L-15 medium was then added, and samples were collected at 2-, 4-, 6-, 12-, and 24-hours post-infection (hpi). Two biological replicates were included for each time point. Collected cells were processed for RNA extraction, cDNA synthesis, and gene expression analysis.

### 2.9 Experimental animals and tissue sampling

Sample Collection. Tissue samples were obtained from twelve clinically healthy Atlantic salmon (Salmo salar, 100 ± 10 g), sourced from the Salmon Clinical Trials Facility at the Universidad Austral de Chile. Fish were acclimated in 1000 L tanks containing UV-treated seawater maintained at 16°C, under a 12-hour light/dark photoperiod. During acclimation, fish were fed a commercial pellet diet at 1% of their biomass (w/w).

Prior to tissue collection, fish were euthanized using an overdose of benzocaine (250 mg/L). Samples were collected from the anterior and posterior kidney, spleen, liver, heart, skeletal muscle, brain, eye, and gills. All samples were preserved in RNAlater (Life Technologies, Carlsbad, CA, USA) and stored at –80 °C until further analysis.

Ethical Approval. All experimental procedures were reviewed and approved by the Bioethics Committee of the Universidad Austral de Chile (Approval No. 440/2021).

### 2.10 Cohabitation challenge with *P. salmonis*

Experimental Setup. A cohabitation challenge was conducted using the *P. salmonis* EM-90-like strain and smolt Atlantic salmon (140–170 g). The experiment was carried out in two 500 L tanks containing filtered UV-treated seawater maintained at 14°C with a salinity of 32 ppt. Each tank housed a total of 120 fish, consisting of: 80 “trojan” fish (66%), which received a 0.1 mL intraperitoneal bacterial inoculum and were marked by caudal fin clipping. 40 healthy cohabitants (33%), which were not injected. Stocking density was maintained below 40 kg/m³. The trial was terminated at 49 days post-inoculation (dpi).

Sampling and Mortality Monitoring. Sampling was conducted on three live or moribund cohabitants at 0, 7, 14, 21, 28, 35, 42, and 49 dpi from one of the two tanks, while the second tank remained unaltered. Mortality was recorded daily. Anterior kidney samples (0.5 cm³) were collected, preserved in RNAlater, and stored at −80°C for further analysis.

Ethical Approval. The experiment was approved by the ADL Bioethics Committee and adhered to the ethical guidelines set by the National Agency for Research and Development (ANID) (accessed on September 4, 2023).

### 2.11 Statistical analyses

Gene expression data from both *in vitro* and *in vivo* experiments were expressed as mean ± standard error (SE). Prior to statistical testing, normality and homogeneity of variances were assessed. *In vitro* group differences were analyzed using one-way analysis of variance (ANOVA) with a significance threshold set at p < 0.05. The *in vivo* assays, where data exhibited non-parametric distributions, the Kruskal-Wallis test was applied, followed by Dunn’s *post hoc* test for multiple comparisons. All statistical analyses were performed using R (v4.3.2) (76). Data visualizations were generated with the ggplot2 package (v3.5.1) (77). Results were considered statistically significant at p < 0.05.

## 3 Results

### 3.1 Identification and comparative analysis of orthologous *sting1* gene in *S. salar*

**Figure 1:**
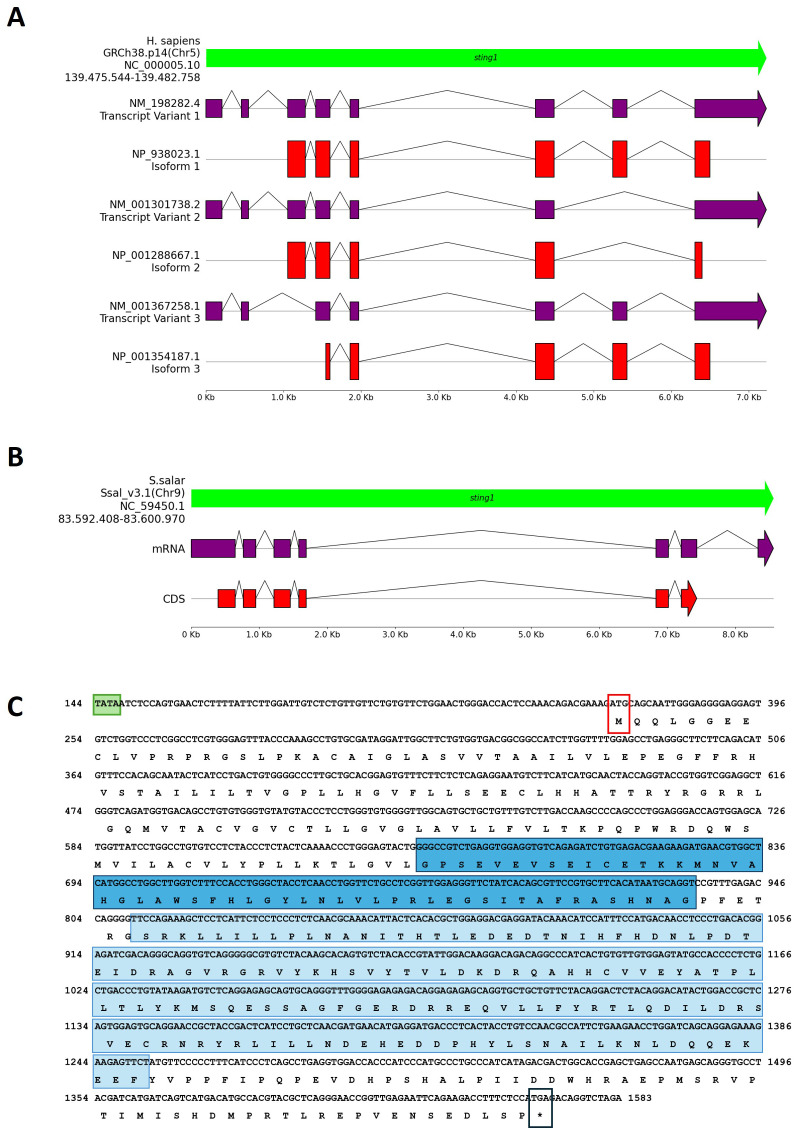
Comparative genomic organization of the sting1 gene in *H. sapiens* and *S. salar*. **(A)** Genomic structure of the *H. sapiens* sting1 locus on chromosome 5 (GRCh38.p14; NC_000005.10), displaying three transcript variants and corresponding isoforms. Exon-intron structures and alternative transcriptional start sites are shown. **(B)** Genomic and transcript structure of *sting1* in *S. salar* (Ssal_v3.1; NC_059450.1), illustrating conservation and divergence relative to the human ortholog. **(C)** Nucleotide and amino acid sequence of *S. salar sting1*, highlighting the TATA box (green), transmembrane (STING-TM, blue) and C-terminal signaling domains (STING-C, light blue), start codon (red box), and stop codon (black box). Colored annotations facilitate identification of functionally relevant regions across the sequence.

Genomic Architecture and Variant Analysis in *H. sapiens*. The *sting1* gene in humans is located on chromosome 5 (GRCh38.p14, NC_000005.10) at approximately 139.5 Mb. It encodes three transcript variants (NP_938023, NP_001288667.1, and NP_001354187.1) with eight, seven, and seven exons, respectively, reflecting differences in exon composition and transcriptional start sites. These isoforms produce proteins of 379, 283, and 260 amino acids (**Figure 1A**). Notably, not all exons contribute to the protein sequence. Transcript variant NP_938023, also identified as *sting1*, aligns with the reference sequence MF622062.1, which is the best-characterized *sting1* sequence in *H. sapiens*. Both NP_938023 and MF622062.1 consist of 1943 nucleotides (nt) and encode a protein of 379 amino acids (aa).

Orthologous *sting1* in *S. salar*. Using the human *sting1* isoform as a reference, a BLAST analysis identified the orthologous *sting1* gene in *S. salar*, a novel finding in fish immunogenetics. The *S. salar sting1* (*Ssa.sting1*) gene contains seven exons (**Figure 1B**), six of which encode a 399-aa protein. The transcript spans 1821 nt and is located on chromosome 9 (Ssal_v3.1, NC_059450.1) between positions 83,592,408 and 83,600,970. The *Ssa.sting1* gene shares ~75% sequence identity with the human isoform 1. Moreover, the result showed that structure differs significantly from the human *sting1*, particularly in exon-intron organization. Additionally, a second *sting*-like sequence was identified on chromosome 5 (Ssal_v3.1, NC_059445.1), between positions 18,599,658 and 18,606,569. However, according to annotations in the NCBI database and SalmoBase (LOC123743239), this locus corresponds to a putative pseudogene.

Genomic Organization and Domain Features in *S. salar*. The **Figure 1C** showed the mRNA and translated protein sequences of STING1 in *S. salar*, detailing the coding DNA sequence (CDS) and highlighting potential functional domains and active sites. Notably, the *Ssa.sting1* gene includes key structural elements such as the TATA box, positioned 96 nucleotides upstream of the start codon, colored in red box, as well as distinct regions sequence that code to the transmembrane domain (STING-TM) and C-terminal (STINGc) domains (indicated by the TATA box in a light green box, methionine start codon in a red box, stop codon in black box, STING-TM coding region in a blue box, and STINGc coding region in a light blue box).

### 3.2 Phylogenetic reconstruction of *sting1* mRNA reveals deep evolutionary conservation across vertebrates

To elucidate the evolutionary relationships of *sting1* across vertebrate taxa, we conducted a comprehensive phylogenetic analysis using full-length mRNA sequences from 39 species representing five major vertebrate classes. Sequences were retrieved from NCBI (accessed January 2024) and are detailed in **Supplementary Table 1**, including GenBank accession numbers and taxonomic classifications.

Multiple sequence alignments were performed using Clustal Omega (42), applying default parameters (gap opening penalty = 6; gap extension = 1; substitution matrix = Gonnet). Ambiguously aligned regions and terminal gaps were manually curated and trimmed to retain only high-confidence blocks of homologous positions suitable for phylogenetic inference.

The phylogenetic tree was generated using the Maximum Likelihood (ML) method implemented in MEGA 11 (43) and exported in Newick format. Tree visualization was performed using the Interactive Tree of Life (iTOL) tool (44), enabling annotation of clade colors and bootstrap values. An unrooted tree topology was selected to display the relationships between taxa rather than the direction of evolutionary time to avoid the imposition of arbitrary ancestral states.

**Figure 2:**
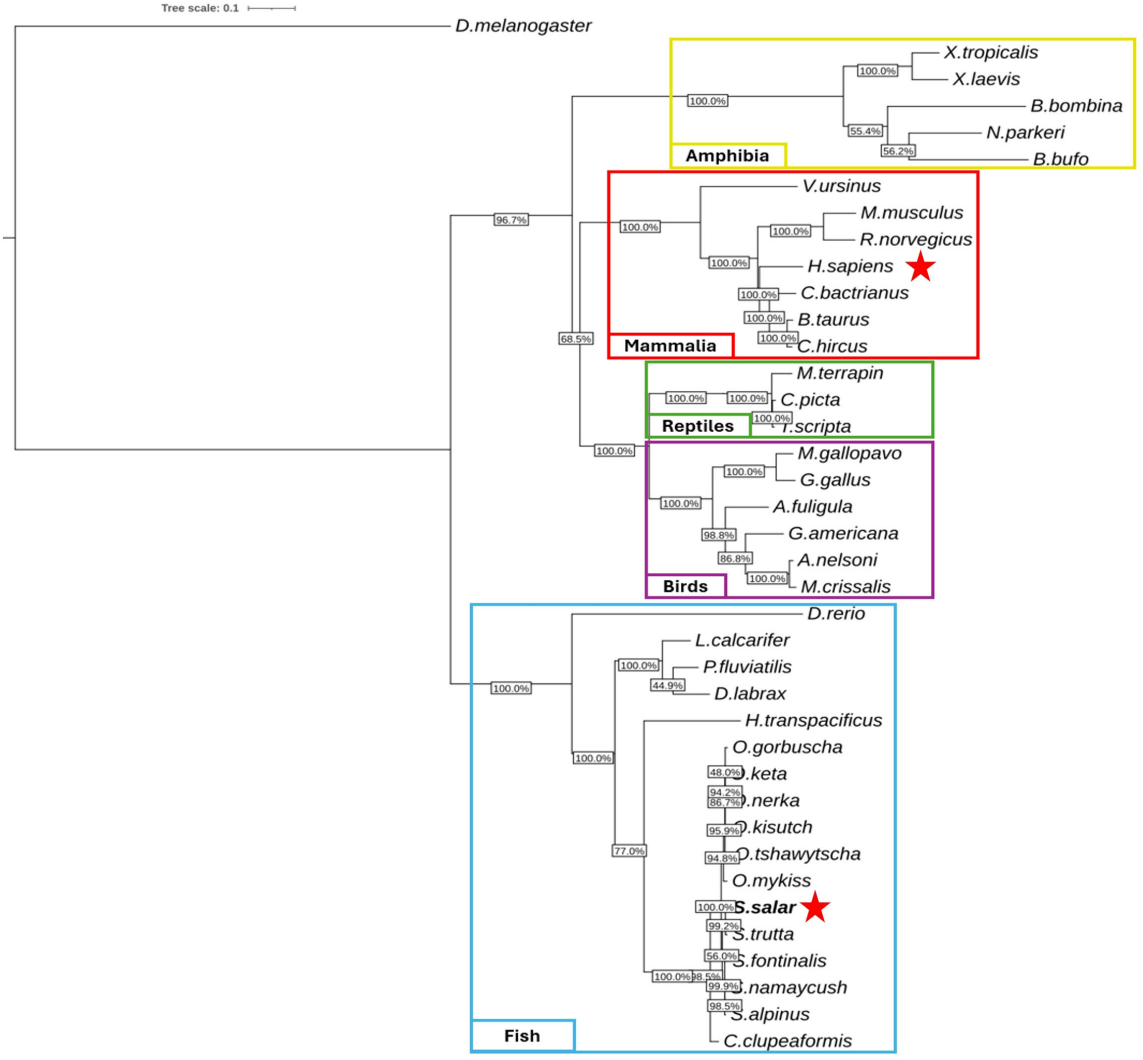
Phylogenetic analysis of *sting1* mRNA sequences across vertebrate lineages. unrooted phylogenetic tree reconstructed using maximum likelihood (10,000 bootstrap replicates), based on *sting1* mRNA sequences from 39 vertebrate species. Taxonomic groups are color-coded: fish (blue), birds (purple), reptiles (green), mammals (red), and amphibians (yellow). *Drosophila melanogaster* was used as an outgroup. Bootstrap values indicate statistical support for branching. Red stars mark the positions of *H. sapiens* and *S. salar*, highlighting evolutionary proximity within their respective clades.

Bootstrap support was calculated using 10,000 replicates (78), and values were mapped onto the branches of the phylogenetic tree (**Figure 2**). Bootstrap values exceeding 70% were interpreted as providing moderate support, while values above 90% were considered strong indicators of phylogenetic confidence. The resulting topology showed robust statistical support for most internal branches.

The unrooted tree delineated five well-defined clades, corresponding to fish (blue), amphibians (yellow), reptiles (green), birds (purple), and mammals (red). Within the teleost fish clade, *Salmo salar* clustered tightly with other salmonids—*Oncorhynchus mykiss* and *O. kisutch*—indicating strong conservation of *sting1* under shared environmental and immunological constraints. Short branch lengths within salmonids suggest recent diversification, whereas longer branches for *Danio rerio* highlight its distinct evolutionary trajectory.

Notably, *D. rerio* was positioned centrally within the unrooted topology, indicating considerable divergence from both salmonids and mammals. The evolutionary distance between *D. rerio* and *S. salar* was comparable to that observed between *D. rerio* and *H. sapiens*, suggesting that zebrafish STING1 has undergone substantial lineage-specific modifications. This divergence may reflect differences in innate immune system architecture or adaptation to distinct ecological pressures.

In contrast, the tight clustering of salmonid species reflects high sequence conservation, potentially driven by persistent exposure to aquatic pathogens and the necessity of maintaining cytosolic DNA sensing integrity in fish. This observation supports the hypothesis that functional conservation of STING1 has been strongly maintained in certain lineages where immune surveillance remains under high evolutionary pressure.

Outside the teleost group, the mammalian clade was supported by bootstrap values >90%, indicating phylogenetic stability and shared ancestry within this group. Interestingly, reptilian and mammalian clades branched adjacently, implying that core STING-mediated signaling pathways may have been established prior to their evolutionary divergence. Avian species formed a separate, well-supported branch, indicative of an independent evolutionary course for innate immune sensors in birds.

The amphibian clade, while distinct, connected to the basal vertebrate node, potentially representing a transitional architecture in early tetrapod evolution. Collectively, the phylogenetic reconstruction supports the conclusion that sting1 is an ancient and deeply conserved immune sensor, whose domain structure and signaling role have been preserved across hundreds of millions of years of vertebrate diversification.

This result reinforces the concept that STING1 plays a central and non-redundant role in cytosolic DNA sensing and innate immunity across diverse taxonomic groups. The detailed evolutionary relationships observed herein offer a foundational framework for future comparative immunology studies and for understanding how STING-driven pathways have been shaped by distinct pathogen landscapes across ecological niches.

### 3.3 Domain architecture and sequence conservation of STING1 homologs across vertebrate lineages

To assess the evolutionary conservation of STING1 architecture across vertebrates, we performed a comparative analysis focusing on the transmembrane (STING-TM) and C-terminal (STINGc) domains. Protein sequences from *S. salar*, *X. maculatus*, *D. rerio*, *X. laevis*, *M. gallopavo*, *M. musculus*, and *H. sapiens* were retrieved and aligned using ClustalW (42). Visual inspection and conservation metrics were obtained via Jalview (51), enabling detailed evaluation of domain conservation, sequence quality, consensus profiles, and occupancy scores. Taxa were selected to represent a broad evolutionary spectrum spanning teleost fish, amphibians, birds, and mammals.

**Figure 3:**
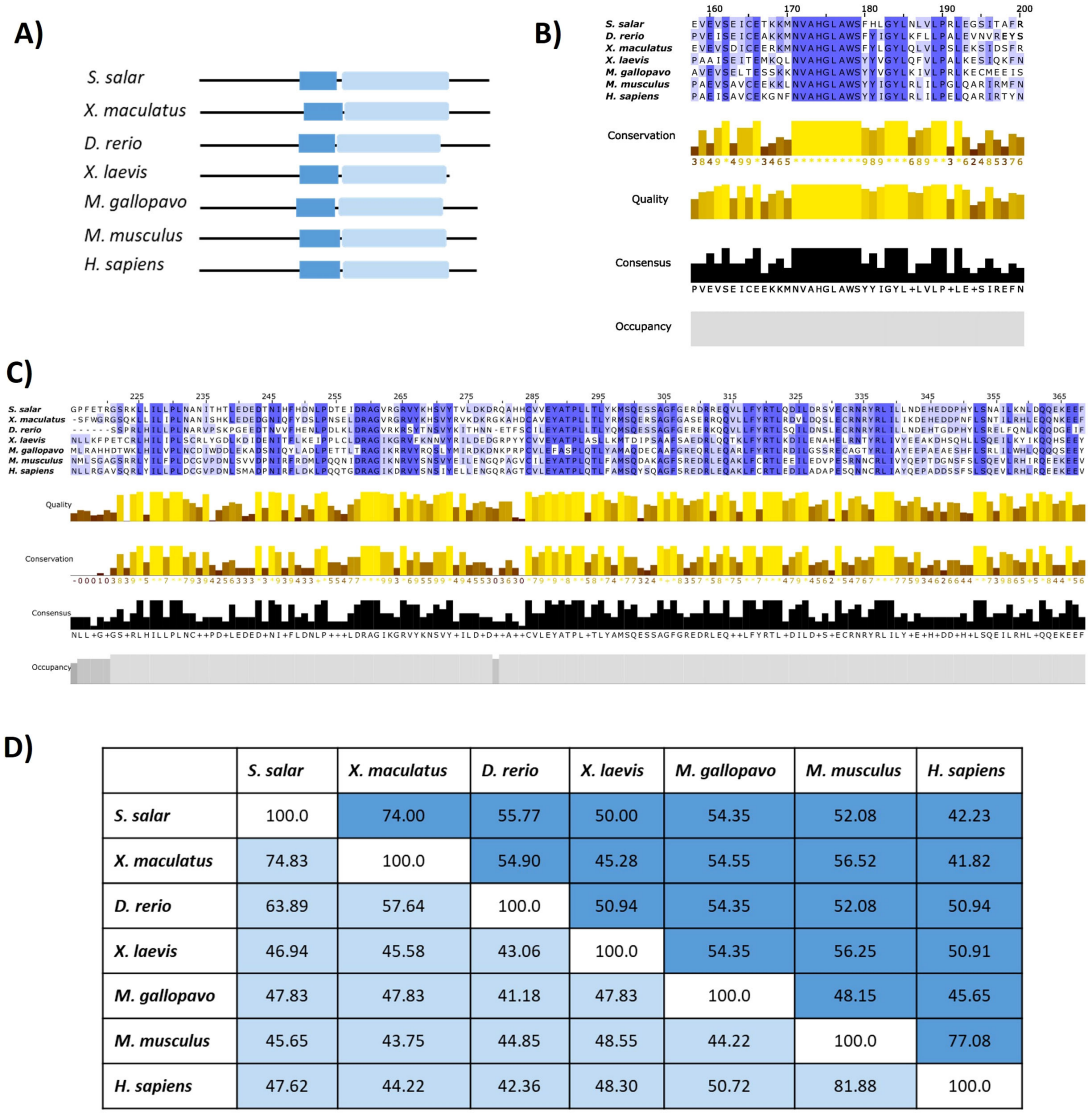
Comparative domain architecture and sequence conservation of STING1 across species. **(A)** Domain schematics showing the conserved transmembrane (STING-TM, blue) and C-terminal signaling (STING-C, light blue) domains across selected species. **(B)** Multiple sequence alignment of the STING-TM domain, with conservation intensity depicted (blue shading). **(C)** Alignment of the STING-C domain, with conservation (yellow shading) and occupancy graphs indicating sequence robustness. **(D)** Similarity percentage matrix comparing transmembrane and C-terminal domains across species, emphasizing evolutionary conservation and divergence patterns.

Domain Mapping Reveals Conserved STING Architecture Across Vertebrates. Domain topology analysis identified two major conserved regions across all species: the STING-TM domain, located near the N-terminus, and the STINGc domain, at the C-terminal region (**Figure 3A**). Although these domains were consistently present, the interdomain spacing varied among taxa, with *S. salar* exhibiting the largest distance. The STING-TM domain remained positionally stable, while the C-terminal segment displayed greater structural divergence, likely reflecting lineage-specific immune adaptations.

High Conservation of the Transmembrane Domain and the Dimerization Motif. Multiple sequence alignment of the STING-TM domain (**Figure 3B**) revealed conserved residues essential for structural integrity and dimerization-dependent activation. The GXXXS motif, a hallmark of STING dimerization critical for downstream recruitment of TBK1 and activation of IRF3 (19), was preserved across all vertebrate taxa analyzed, including *S. salar*. Additional residues—S162, E166, L189, P190, L192, and the conserved NVAHGLAWS motif (residues 171–179 in *S. salar*)—exhibited high conservation, underscoring their functional relevance in stabilizing membrane topology and protein folding. Conservation was further corroborated by high-quality scores, occupancy graphs, and consensus profiles, reflecting evolutionary constraints across teleosts, amphibians, and mammals.

Conservation of the C-Terminal Signaling Domain Across Species, alignment of the STINGc domain (**Figure 3C**) revealed substantial conservation in residues associated with signal transduction, particularly those involved in TBK1 binding and downstream immune activation. Physicochemical conservation metrics confirmed the preservation of core residues across species, supporting the functional resilience of STING’s C-terminal module. Divergences observed in peripheral regions of the domain likely reflect clade-specific adaptations to distinct pathogen pressures or immune niches.

Quantitative Assessment of Cross-Species Similarity: Pairwise similarity analyses (**Figure 3D**) provided quantitative support for the observed structural conservation. The STING-TM domain displayed higher similarity percentages across taxa relative to the STINGc domain, suggesting tighter functional constraints on membrane anchoring and dimerization. Between *S. salar* and *H. sapiens*, the STING-TM domain shared 44.23% sequence identity, while the STINGc domain exhibited 47.62% similarity. This pattern supports a model of domain-specific evolutionary pressure, wherein essential motifs are preserved to maintain core functionality, while flexible regions permit adaptive divergence.

Comparative insights between *S. salar* and *H. sapiens*, although overall sequence identity between *S. salar* and *H. sapiens* remains moderate, critical residues required for membrane insertion, dimerization, and signal propagation are conserved. The maintenance of the GXXXS motif and cysteine-rich subregions in *S. salar* suggests functional retention of STING’s immune-sensing capacity. These findings highlight the deep evolutionary conservation of the STING pathway as a central node in vertebrate cytosolic DNA sensing and innate immune activation, reaffirming the role of STING as an evolutionarily entrenched immune integrator.

### 3.4 Comparative protein modeling and molecular docking analysis

**Figure 4:**
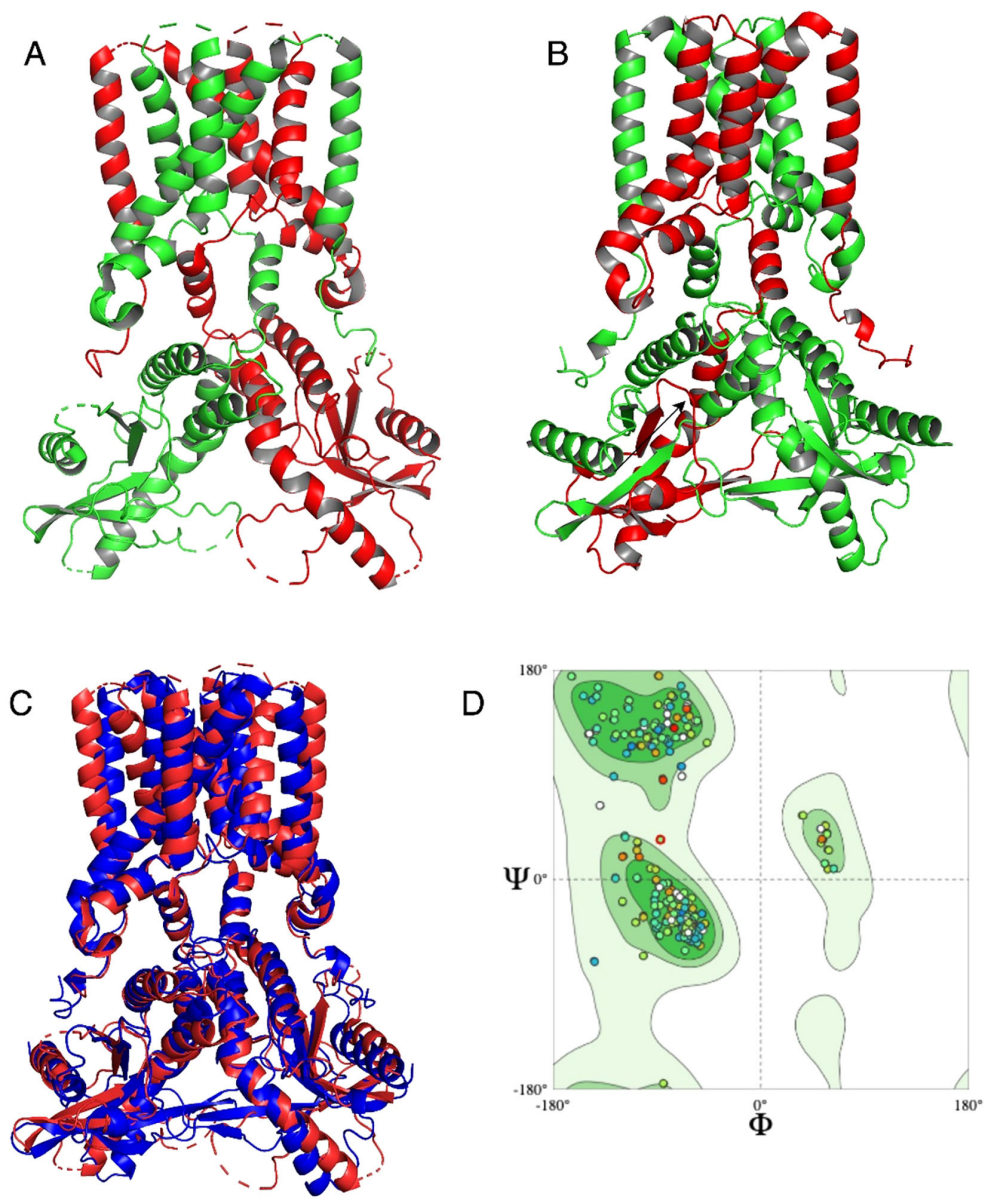
Structural alignment and quality assessment of the predicted Ssa.STING1 model. **(A, B)** Structural superposition of the predicted *S. salar* STING1 (green) and the crystallographic *H. sapiens* STING (red), highlighting overall architecture conservation. **(C)** Focused comparison of binding pocket regions between the two models. **(D)** Ramachandran plot analysis of Ssa.STING1 validating stereochemical quality, with a high percentage of residues in favored conformational spaces.

Three-dimensional structures of the STING protein were predicted using AlphaFold3. Validation of the structural quality of the predicted *S. salar* STING1 (SSa-STING1) model via a Ramachandran plot revealed that 88.66% of the residues fell within favored regions, indicating a high-quality prediction. Structural alignment between the SSa-STING1model and the native crystal structure of *H. sapiens* STING showed a significant overlap, with a Root Mean Square Deviation (RMSD) of 2.218 Å, highlighting the structural similarity and consistency between the two proteins (**Figures 4A, B**).

Statistical validation further substantiated the accuracy of the predicted models. The ERRAT score, which evaluates the quality of non-bonded atomic interactions, confirmed robust structural integrity for both proteins, with *H. sapiens* exhibiting slightly better scores than *S. salar*. VERIFY3D assessments, evaluating atomic model compatibility with the amino acid sequence, yielded scores of 52.77% for *H. sapiens* and 45.61% for *S. salar*. While these values are below the optimal threshold, PROCHECK analysis verified that the stereochemical quality of both models was reliable, with minimal structural errors. These findings highlight subtle structural variations between the two species, which may underline differences in STING-mediated signaling pathways, while maintaining overall structural integrity. Notably, the conserved alignment in the binding site region (**Figure 4C**) supports functional similarity between the species. The Ramachandran plot of the *S. salar* model (**Figure 4D**) further confirmed the stereochemical reliability, with a high proportion of φ and ψ angles in allowed regions.

**Figure 5:**
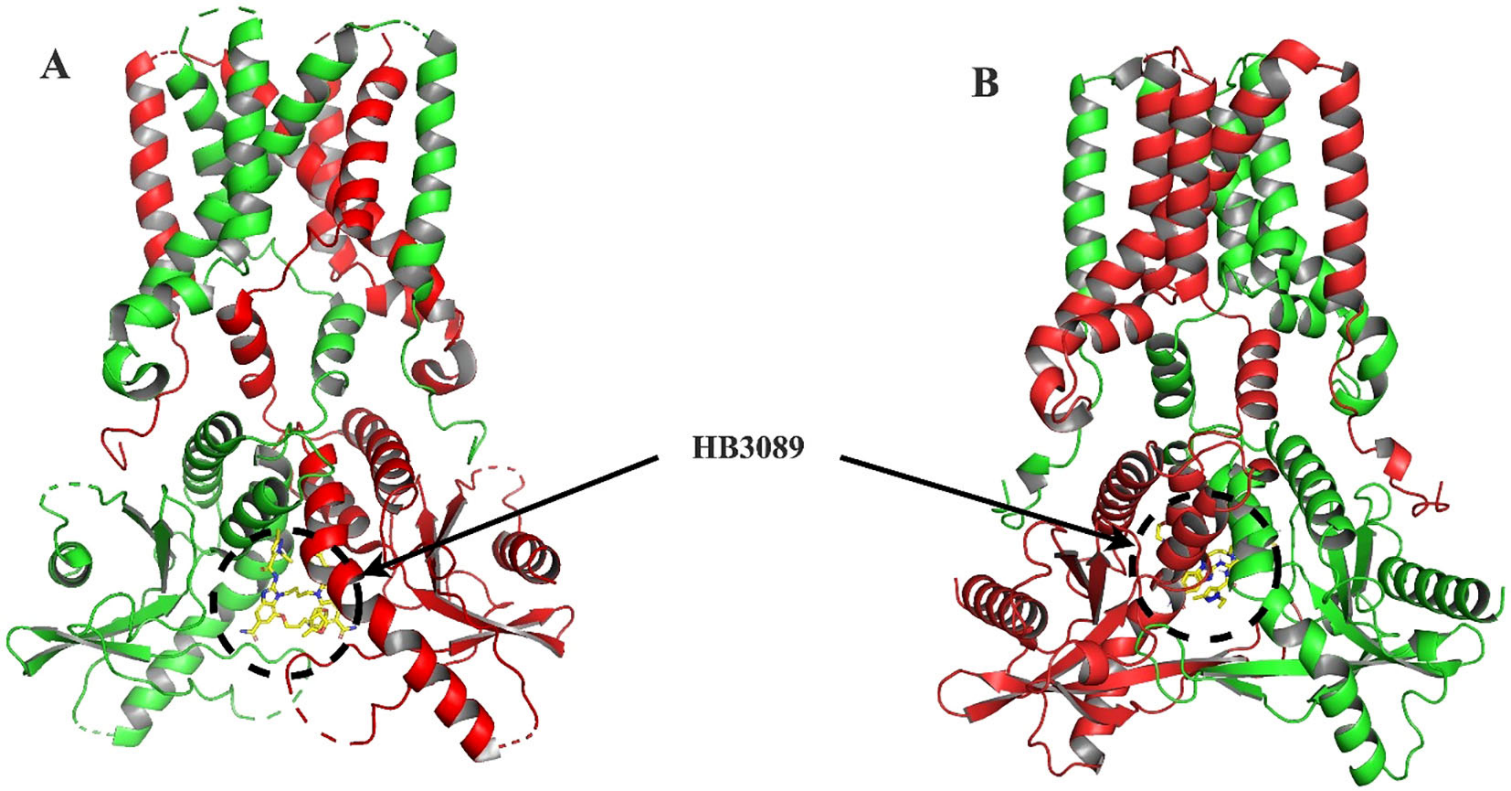
Molecular docking of HB3089 with STING proteins. **(A)** Front view of the molecular docking between the HB3089 agonist (yellow sticks) and the crystallographic structure of *H. sapiens* STING (red), highlighting the ligand’s placement in the native binding pocket. **(B)** Molecular docking results of the same HB3089 ligand against the S. salar STING1 model (green), obtained using AlphaFold 3, illustrating the ligand bound in an analogous region. The comparison between both docking setups shows that, while the ligand occupies a similar spatial location, differences in binding affinity values (-10.2 kcal/mol for *H. sapiens* vs. -6.6 kcal/mol for *S. salar*) suggest evolutionary variations in the binding pocket that may influence the stability and recognition of the agonist.

**Figure 6:**
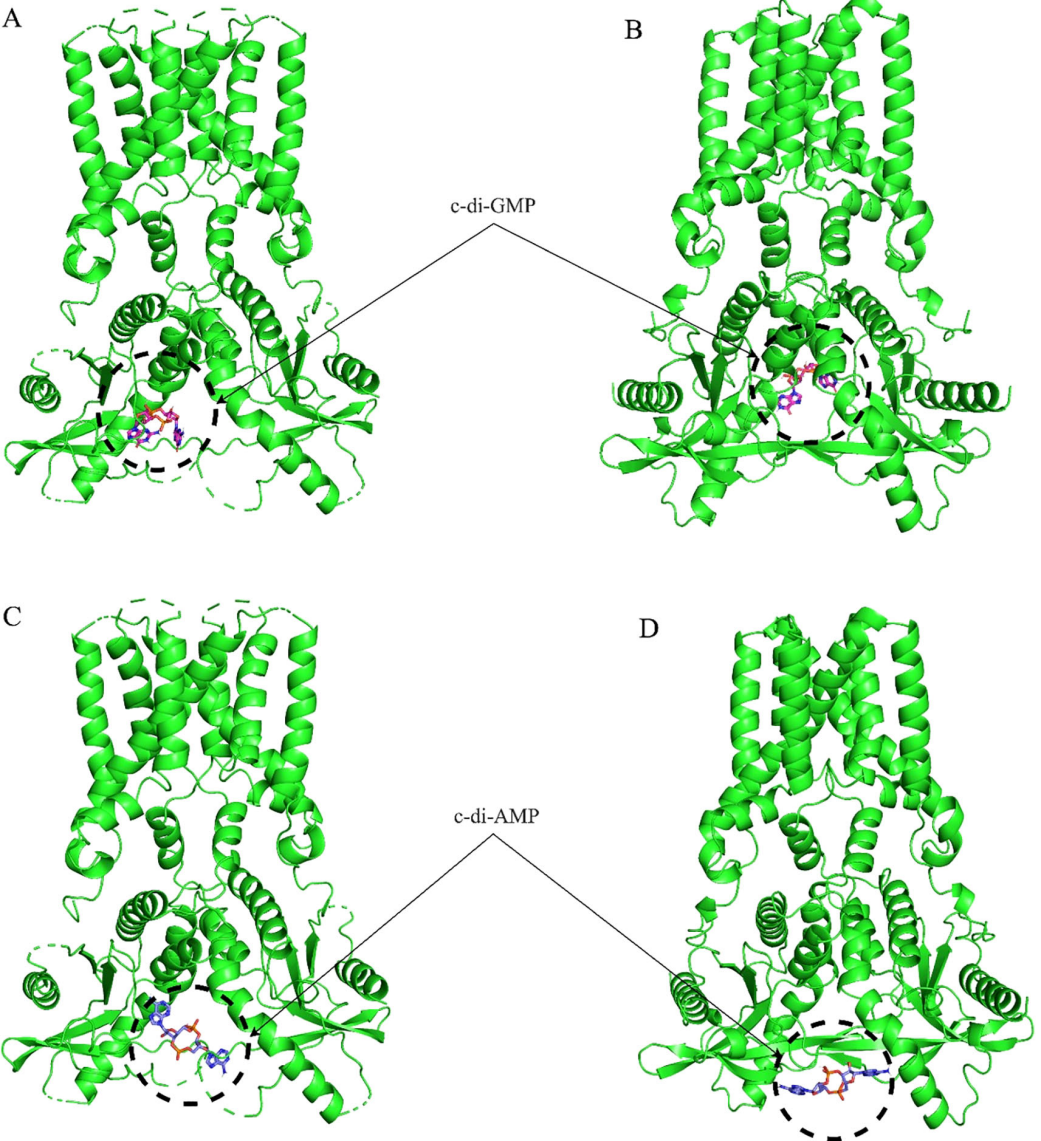
Molecular docking of c-di-GMP and c-di-AMP with STING proteins. **(A)** Frontal view of the molecular docking between c-di-GMP and the crystallographic structure of H sapiens STING (green), showing the ligand positioned in the native binding pocket. **(B)** Molecular docking of the same c-di-GMP ligand with the *S. salar* STING1 model (green), obtained using AlphaFold 3, highlighting the ligand’s location in the analogous binding region. These results illustrate the conservation of the binding site between both species, as well as differences in ligand affinity. **(C)** Frontal view of the molecular docking between c-di-AMP and the crystallographic structure of *H*. *sapiens* STING (green), demonstrating the ligand’s placement in the native binding pocket. **(D)** Molecular docking of the same c-di-AMP ligand with the *S. salar* STING1 model (green), obtained using AlphaFold 3, highlighting the ligand’s position in the analogous binding site. These representations illustrate the similarity in ligand location between both species.

Molecular docking analysis with the HB3089 agonist revealed binding affinities of -6.6 kcal/mol for *S. salar* and -10.2 kcal/mol for *H. sapiens* (**Figure 5**, **Table 2**). Docking with the c-di-GMP ligand resulted in binding affinities of -8.5 kcal/mol for *S. salar* and -10.5 kcal/mol for *H. sapiens* (**Figures 6A, B**), while for the c-di-AMP ligand, affinities were -7.9 kcal/mol for *S. salar* and -9.5 kcal/mol for *H. sapiens* (**Figures 6C, D**). These results suggest evolutionary adaptations in the binding pocket of *S. salar* STING, which may influence ligand stability and recognition compared to *H. sapiens* STING.

**Table 2:** Binding affinity values between STING receptors and ligands determined by molecular docking.

Ligand	*H. sapiens* STING (kcal/mol)	*S. salar* STING1 (kcal/mol)
HB3089	-10.2	-6.6
c-di-GMP	-10.5	-8.5
c-di-AMP	-9.5	-7.9

Binding energies (expressed in kcal/mol) of the interaction between human (*H. sapiens*) STING and salmonid (*S. salar*) STING1 receptors with three ligands: HB3089, c-di-GMP, and c-di-AMP. Docking analyses were conducted using AutoDock Vina. More negative values indicate stronger predicted binding affinities.

### 3.5 Expression pattern of the STING gene in healthy tissues of *S. salar*

**Figure 7:**
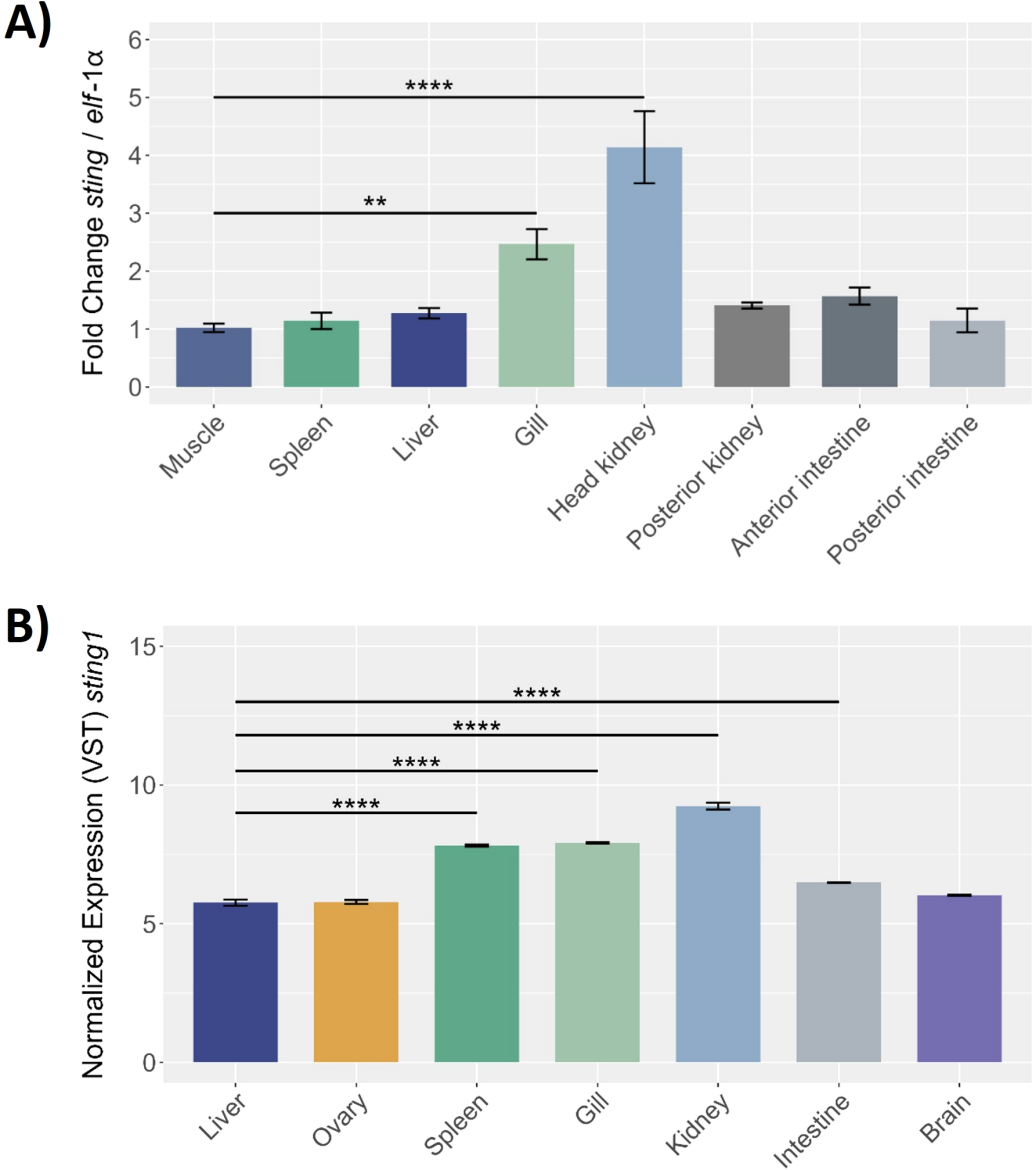
Sting gene expression in different healthy tissues of *S. salar* assessed by **(A)** RT-qPCR and **(B)** RNA-seq. The RT-qPCR expression data were normalized to elongation factor-1α (*elf*-1α) levels, with the spleen serving as the control tissue. The RNA-seq of control condition of *S. salar*, retrieved from public databases using the SRA Toolkit. Sample data in triplicate for each tissue, normalized data by VST. Statistical with One-way ANOVA, and *post-hoc* with Tukey pairwise comparison. Values are presented as mean ± SE, with statistical significance indicated by asterisks: (**) p < 0.01; (****) p < 0.0001.

This study evaluated *Ssa.sting1* gene expression across various healthy tissues in *S. salar*. Initially, the expression levels were quantified by RT-qPCR and normalized to *elf*-1α in eight tissues: muscle, spleen, liver, gill, head kidney, posterior kidney, anterior and posterior intestine. *Ssa.sting1* was expressed in all tissues, with the lowest levels in muscle, which served as the baseline for comparisons. The highest expression was observed in the head kidney, followed by the gill, showing 4- and over 2-fold increases relative to muscle (p < 0.0001 and p < 0.01, respectively) (**Figure 7A**).

RNA-seq data from the NCBI database, under control conditions, corroborated these findings, revealing significant *Ssa.sting1* expression in kidney, gill, spleen, liver, intestine, brain, and ovary, with notably high levels in kidney, gill, and spleen (**Figure 7B**). These results represent the first report of constitutive *sting1* expression across a broad range of tissues in *S. salar*. Elevated expression in the kidney, gills, and spleen suggests a crucial role in physiological and immunological processes. Tissue-specific variations in *sting1* expression highlight its potential involvement in modulating immune responses and maintaining homeostasis in diverse biological contexts.

### 3.6 Gene expression levels of STING during *P. salmonis* infection in SHK-1 cells

**Figure 8:**
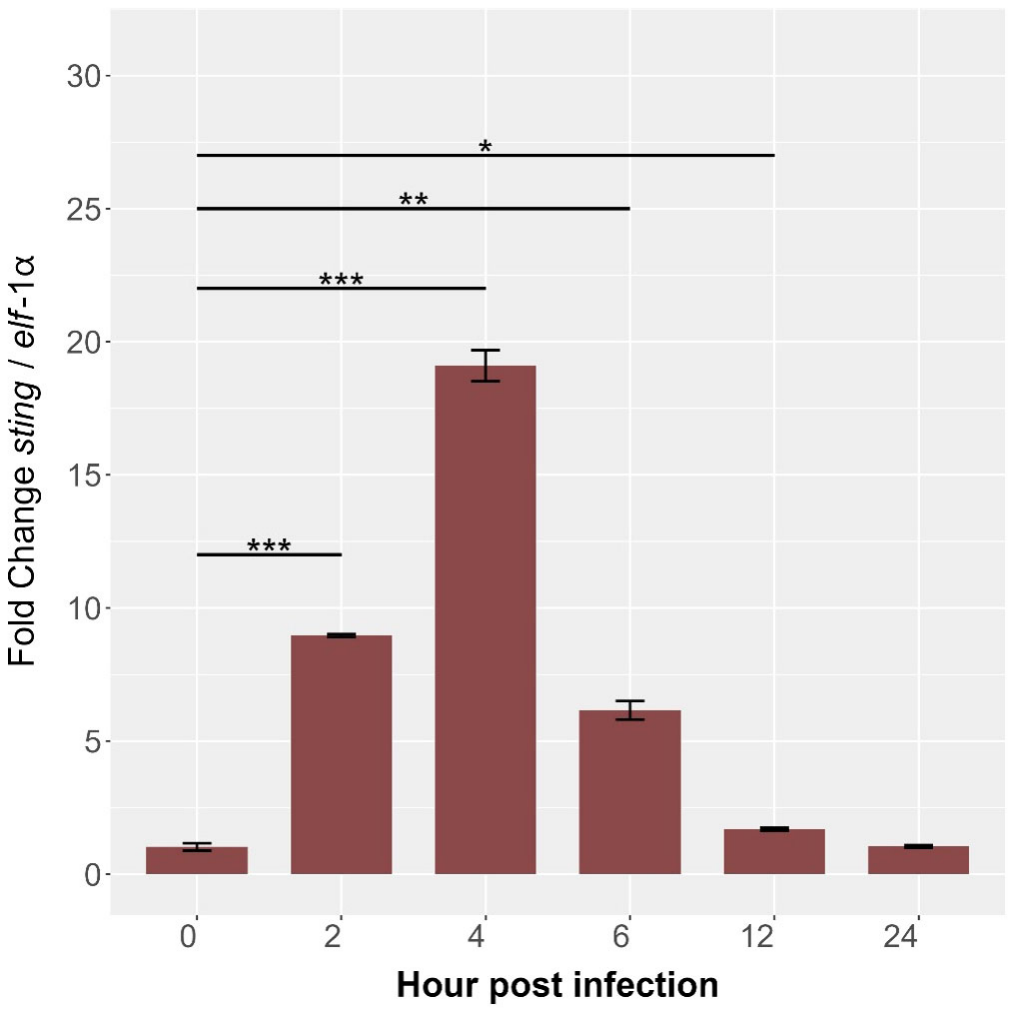
Kinetics of gene expression in the *Ssa.sting1* from 0 (control) to 24 hpi in SHK-1 cells infected with *P. salmonis*. Gene expression levels were normalized to elongation factor-1α (*elf*-1a) using qRT-PCR. Data are presented as mean ± SE, with statistical significance indicated by asterisks: (*) p < 0.05; (**) p < 0.01; (***) p < 0.001 compared to control (non-infected SHK-1 cells).

Quantitative PCR analysis revealed dynamic *sting1* expression in SHK-1 cells during *P. salmonis* infection, highlighting its role in the immune response of *S. salar*. At baseline (0 hours), expression was minimal. Post-infection, *Ssa.sting1* expression increased 8-fold by 2 hours and peaked at nearly 18-fold by 4 hours (p < 0.01) (**Figure 8**). Afterward, expression declined to 11-fold at 6 hours and 7-fold at 12 hours, reaching 5-fold above baseline by 24 hours (p < 0.0001). This temporal decline may indicate an adaptation phase as the cells modulate their response to the persistent presence of the pathogen. These findings underscore the pivotal role of STING in orchestrating early immune signaling pathways in response to *P. salmonis* infection, offering valuable insights into the transcriptional regulation and functional dynamics of the STING pathway under pathogenic stress in aquaculture environments.

### 3.7 Expression patterns of immune-related genes in the STING pathway during *P. salmonis* infection in SHK-1 cells

**Figure 9:**
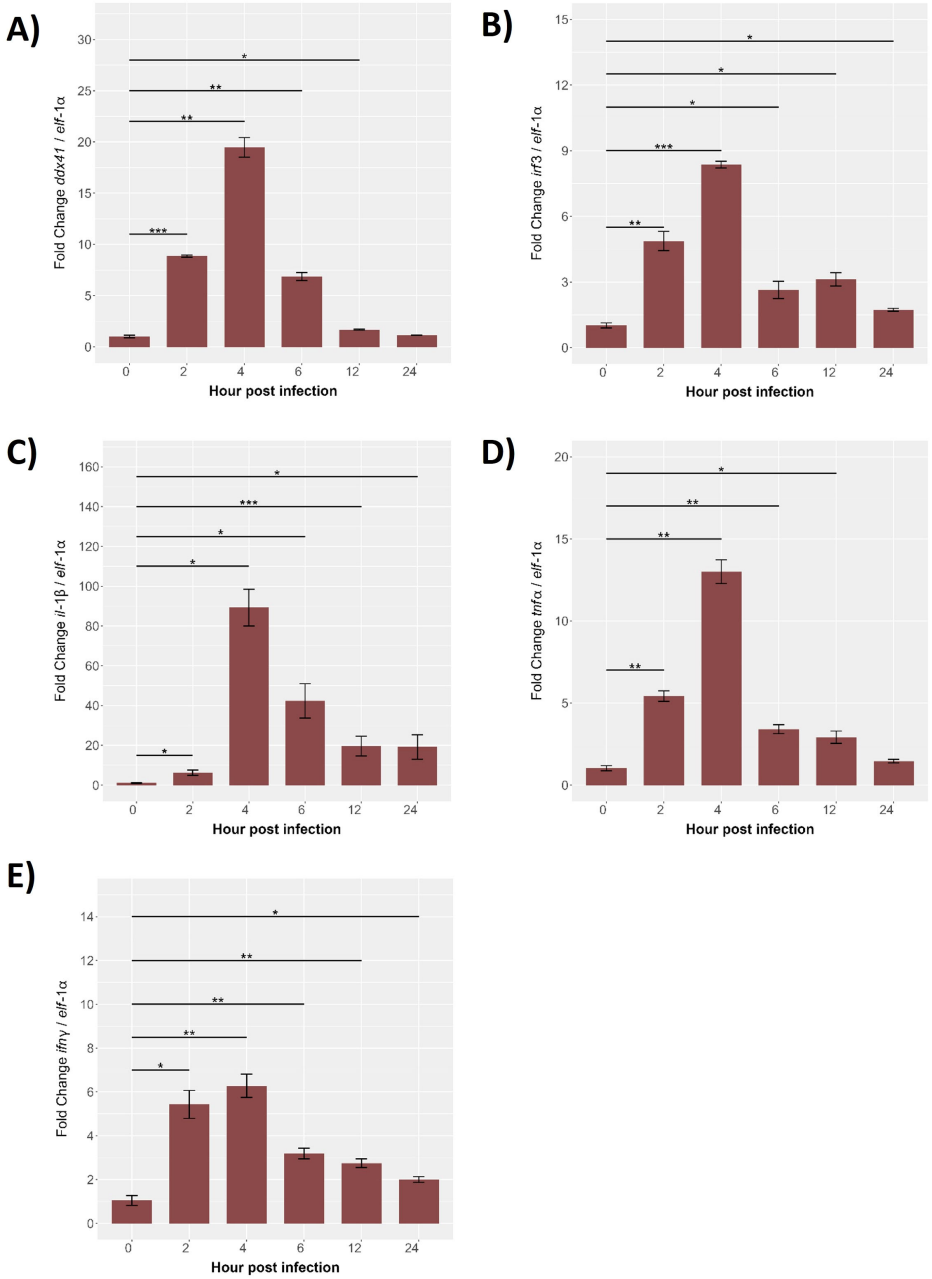
Gene expression kinetics of the innate immune response from 0 (control) to 24 hpi in SHK-1 cells Infected with *P. salmonis*. Gene expression levels of **(A)** *ddx41*, **(B)** *irf3*, **(C)** *il*-1β, **(D)** *tnf*-α and **(E)** *ifn*-γ were assessed via RT-qPCR. Expression was normalized to elongation factor-1α (*elf*-1α) using qRT-PCR. Data are presented as mean ± SE, with statistical significance indicated by asterisks: (*) p < 0.05; (**) p < 0.01; (***) p < 0.001 compared to control (non-infected SHK-1 cells).

The temporal expression of immune-related genes (*ddx41*, *irf3*, *il*-1β, *tnf*-α, and *ifn*-γ) was analyzed in SHK-1 cells during *P. salmonis* infection (0–24 hours), normalized to *elf*-1α. The *ddx41* expression was low at baseline but increased significantly by 2 hours, peaking at 25-fold by 4 hours (p < 0.01), followed by a gradual decline to near-baseline levels by 24 hours (**Figure 9A**). Similarly, *irf3* peaked at 4 hours with an 8-fold increase (p < 0.001) before progressively declining to baseline by 24 hours (**Figure 9B**). The most pronounced response was observed for *il*-1β, which peaked at 100-fold above baseline by 4 hours (p < 0.001), gradually declining but remaining significantly elevated at later time points (**Figure 9C**). The *tnf*-α expression increased 2-fold by 2 hours, peaking at 15-fold by 4 hours (p < 0.01), and gradually declined thereafter (**Figure 9D**). The *ifn*-γ showed modest increases at 2 hours, peaking at 6-fold by 4 hours (p < 0.001), and returned to near-baseline levels by 24 hours (**Figure 9E**). These significant temporal changes in expression for all genes examined underscore their critical roles in mediating the immune response of *S. salar* to *P. salmonis* infection. The observed patterns—characterized by early induction, peak expression, and subsequent downregulation—highlight the involvement of these genes in orchestrating the host’s response from initial activation to resolution, providing valuable insights into the regulatory mechanisms of fish immunity.

### 3.8 STING expression during cohabitant challenge with *P. salmonis*

**Figure 10:**
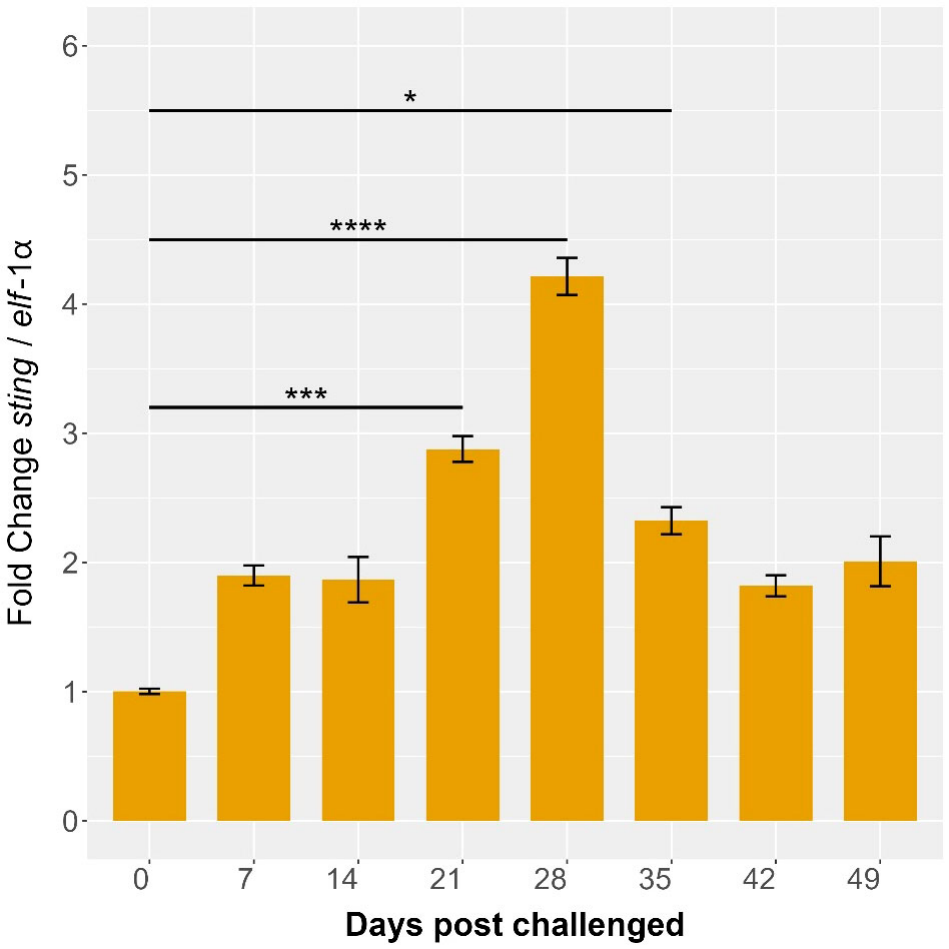
*Ssa.sting1* expression during cohabitant challenge of *S. salar* with *P. salmonis*. The cohabiting (naive) group exhibited mortalities beginning at 28 days post-infection (dpi), reaching 70% by 49 dpi. Gene expression levels of sting were normalized to *elf*-1α levels. Values are presented as mean ± SE, with statistical significance indicated by asterisks: (*) p < 0.05; (***) p < 0.001; (****) p < 0.0001 compared to the uninfected *S. salar* control.

The expression levels of the *Ssa*-*sting1* gene in *S. salar* were monitored over several weeks during a cohabitation challenge with *P. salmonis*. Sampling was conducted at 0-, 7-, 14-, 21-, 28-, 35-, 42-, and 49-days post-infection, with expression normalized to *elf*-1α levels. At baseline (0 days), *Ssa-sting1* expression was minimal, serving as the reference point for subsequent measurements. By 7 days post-infection, a moderate increase in *Ssa-sting1* expression was observed, reaching approximately 1.5-fold relative to baseline (**Figure 10**). This was followed by a slight decrease at 14 days post-infection, with expression levels around 1.2-fold of the baseline (**Figure 10**), suggesting a maintained but moderate immune response as the host interacts with the pathogen.

*Ssa.sting1* expression showed a marked peak at 21 days post-infection, reaching approximately 4.5-fold above baseline (p < 0.05), indicating a robust activation of the immune response, potentially due to increased pathogen load or heightened immune activity. This elevated expression was sustained at 28 days post-infection, with a fold change of about 5 times the baseline (p < 0.01), representing the highest expression level observed during the study period (**Figure 10**). This sustained peak suggests a prolonged immune challenge posed by *P. salmonis*, with continuous activation of the STING pathway.

By 35 days post-infection, *Ssa-sting1* expression began to decline, reaching approximately 3-fold above baseline. This decrease may reflect an adaptation or regulatory adjustment in the immune response as the host works to control the infection. At 42- and 49-days post-infection, STING expression further decreased to around 2.5-fold of baseline levels, with significant reduction observed at 49 days (p < 0.05) (**Figure 10**), indicating a continued resolution of the immune response as the host stabilizes.

The observed increase and decrease in *Ssa-sting1* expression during the cohabitation challenge with *P. salmonis* underscore its critical role in the immune response of *S. salar*. The initial increase, significant peaks at 21 and 28 days, and subsequent decline highlights the gene’s involvement from early activation and sustained immune engagement through to eventual resolution. These findings offer valuable insights into the transcriptional regulation and functional dynamics of the STING pathway during pathogen challenges in aquaculture, emphasizing its importance in mediating immune responses in *S. salar*.

### 3.9 Expression patterns of proinflammatory genes in the head kidney during cohabitant challenge

**Figure 11:**
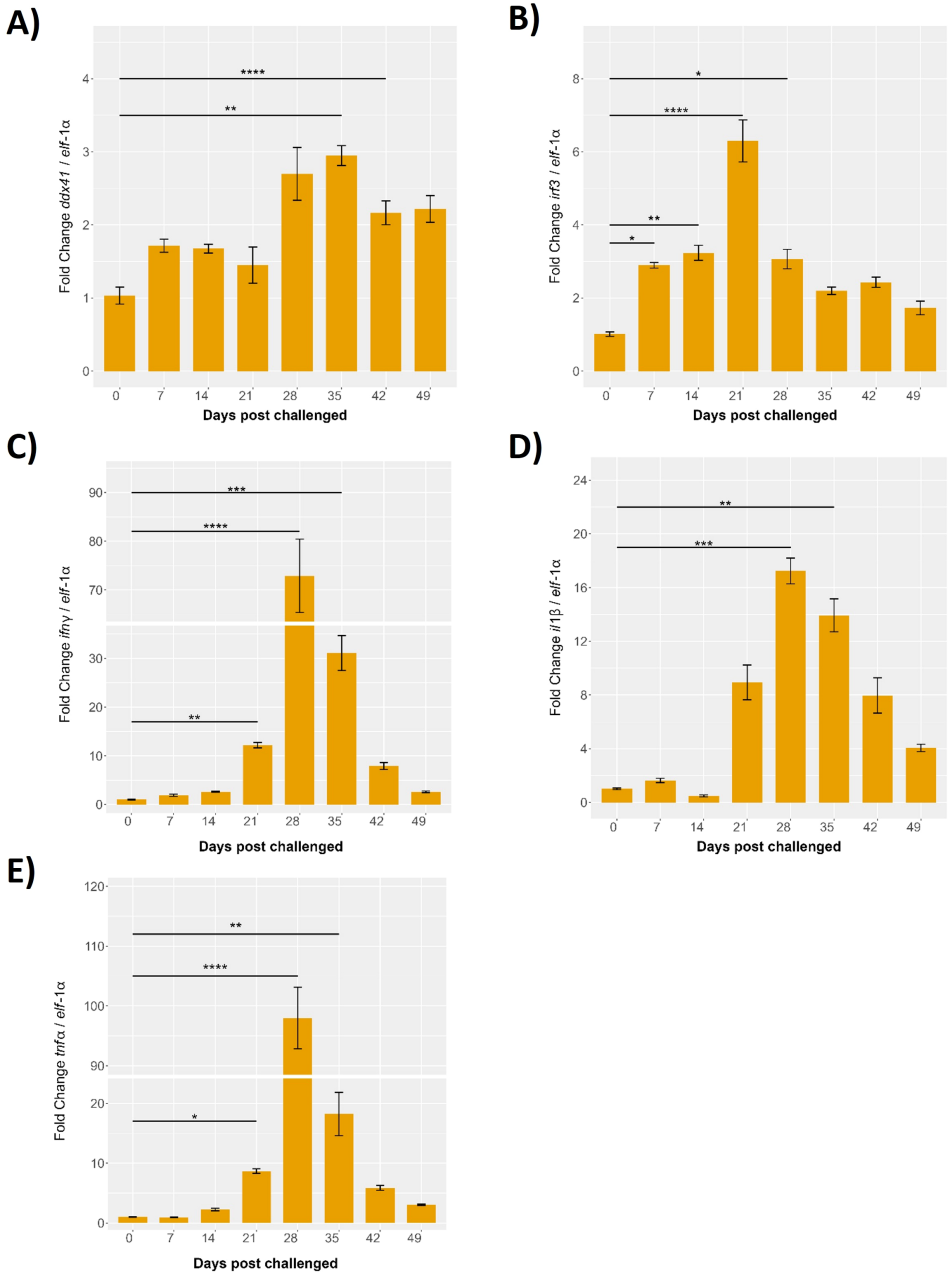
Evaluation of the innate response during cohabitant challenge of *S. salar* with *P. salmonis.* The cohabiting (naive) group exhibited mortalities beginning at 28 days post-challenge (dpc), reaching 70% by 49 dpc. Gene expression levels of **(A)** *ddx41*, **(B)** *irf3*, **(C)** *ifnγ*, **(D)** *il*-1β and **(E)** *tnf*-α were normalized to elongation factor-1α (*elf*-1a) levels. Results are presented as mean ± SE, with statistical significance indicated by asterisks: (*) p < 0.05; (**) p < 0.01; (***) p < 0.001; (****) p < 0.0001 compared to the uninfected *S. salar* control.

The expression levels of key proinflammatory genes in the head kidney of *S. salar* were evaluated over a 49-day period during a cohabitation challenge with *P. salmonis*. The results, normalized to *elf*-1α expression, reveal dynamic changes in gene expression post-infection, underscoring the immune response dynamics in this critical organ. Specifically, the expression levels of immune-related genes associated with the STING pathway *ddx41*, *irf3*, *il*-1β, *tnf*-α, and *ifn*-γ were monitored. At baseline (0 days), expression levels for all genes were minimal. By 7 days post-infection, *ddx41* showed slight increases, indicating initial immune activation, with significant peaks observed at 28 days, reaching approximately 3.5-fold (p < 0.001) compared to baseline. The highest expression levels were recorded at 35 days post-infection, with fold changes of approximately 4 for *ddx41* (p < 0.01), followed by a gradual decline at 42 and 49 days, though levels remained significantly above baseline (p < 0.05) (**Figure 11A**).

The *irf3* expression displayed a modest increase at 7 days post-infection, peaking significantly at 21 days with an 8-fold increase over baseline (p < 0.01). This was followed by a reduction at 28 days, to around 4-fold (p < 0.05), and stabilization at 35 days with a 3-fold increase over baseline (p < 0.01). Expression levels at 42 and 49 days remained slightly elevated relative to baseline, with fold changes of approximately 2-fold, indicating a sustained but moderate immune response (**Figure 11B**).

Gene expression of *il*-1β was minimal until 14 days post-infection, followed by a significant increase at 21 days, reaching approximately 10-fold over baseline (p < 0.05). Peaks were observed at 28 and 35 days, with fold changes of 15 and 12 times the baseline, respectively (p < 0.01 and p < 0.001). Expression levels declined at 42 and 49 days but remained significantly higher than baseline, with fold changes of about 8 and 5 times, respectively (p < 0.01) (**Figure 11C**).

The *tnf*-α expression remained low up to 14 days post-infection, followed by a sharp and significant peak at 28 days, showing a 100-fold increase over baseline (p < 0.01), the highest among the genes studied. This was followed by a decline at 35 days to about 20-fold above baseline (p < 0.01), with slightly elevated levels persisting at 42 and 49 days, showing fold changes of around 15 and 10 times the baseline, respectively (p < 0.01) (**Figure 11D**).

Finally, *ifn*-γ exhibited minor increases at 7- and 14-days post-infection, with a significant peak at 28 days, reaching approximately 100-fold over baseline (p < 0.001). A decrease was observed at 35 days, with a fold change of about 40 (p < 0.01), and slight elevation at 42 and 49 days, showing fold changes of around 20 and 10 times the baseline, respectively (p < 0.001) (**Figure 11E**).

These results highlight the dynamic roles of *Ssa.sting1*, *ddx41*, *irf3*, *il*-1β, *tnf*-α, and *ifn*-γ in immune regulation during *P. salmonis* infection. The observed early increases, significant peaks at distinct time points, and subsequent declines highlight the temporal regulation of these genes and their involvement in orchestrating the immune response. These findings provide valuable insights into the transcriptional regulation and functional dynamics of these immune-related genes during pathogen exposure in aquaculture, emphasizing their importance in the immune defense mechanisms of *S. salar*.

## 4 Discussion

The STING (Stimulator of Interferon Genes) pathway stands among the most ancient and evolutionary conserved architectures of innate immunity across vertebrates. It functions as a central cytosolic sensor and executor—decoding pathogenic nucleic acid signatures into orchestrated immune responses, including type I interferon (IFN-I) induction, inflammatory cytokine secretion, and immunogenic cell death (5, 79, 80). In this study, we provide the first comprehensive structural and functional characterization of the STING ortholog (*Ssa.sting1*) in *Salmo salar*, revealing that—despite more than 400 million years of vertebrate diversification—the STING domain architecture remains deeply conserved across lineages, including the last ∼80 million years of salmonid evolution (7, 54, 81, 82).

Our data confirm that the architecture of the ligand-binding cleft in Ssa.STING1 has undergone strong purifying selection, retaining its capacity to detect both microbial CDNs (c-di-GMP, c-di-AMP) and endogenous cGAMP, the second messenger synthesized by cGAS upon cytosolic DNA detection (83, 84). This evolutionary resilience reflects not static conservation, but a deeply encoded molecular logic of survival, whereby STING operates as a recursive interpreter of infection and damage.

While this structural preservation confirms STING’s ancestral role as an immune transducer, subtle conformational divergences within key residues suggest the emergence of species-specific functional modulations, particularly in aquatic vertebrates exposed to distinct microbial ecologies (85, 86). Docking simulations support this nuance, revealing that although the CDN-binding pocket remains constrained, localized structural shifts may fine-tune activation thresholds in *S. salar*, embodying an evolutionary tension between structural integrity and adaptive specialization (87, 88).

In *S. salar*, we observed a temporally dynamic regulation of sting1, alongside downstream effectors such as *irf3* and *ifn*-β, in response to the facultative intracellular pathogen *P. salmonis*. STING expression surged early post-infection, indicative of a frontline response to pathogen-associated molecular patterns (PAMPs), triggering IFN signaling. However, this induction faded by day 5, despite persistent infection, unveiling a likely immune tolerance or bacterial subversion mechanism.

This response mirrors the biphasic immune kinetics seen in chronic viral infections, wherein initial immune engagement is followed by regulatory silencing, often exploited by pathogens to establish persistence (86, 89). These patterns suggest that STING, rather than serving as a constant amplifier of innate responses, may act as a context-sensitive switch—one modulated not just by pathogen load, but by feedback from stress, damage, and metabolic constraints.

Such temporal regulation underscores an emerging paradigm in which STING functions not merely as a sentinel but as a gatekeeper of immune equilibrium. In this model, intracellular bacteria may downregulate STING activation via multi-layered suppression mechanisms, including transcriptional repression of TMEM173, disruption of Golgi trafficking, and proteasomal degradation mediated by host E3 ligases such as RNF5 or TRIM29 (90, 91).

This temporal regulation supports the emerging view of STING not as a binary immune trigger, but as a contextual integrator of immune fate, capable of balancing activation and restraint according to pathogen strategy, metabolic status, and cellular stress. The downregulation of *ifn*-β and *irf3*, despite sustained *sting1* expression in *S. salar*, suggests a decoupling between pathogen detection and effector output, a phenomenon increasingly recognized in chronic infections and tumor microenvironments.

Pathogens appear to exploit this regulatory complexity. In bacteria like *P. salmonis*, potential immune evasion may involve a convergence of suppression strategies, including epigenetic silencing of TMEM173 (the STING-encoding gene), interference with ER-to-Golgi trafficking, and ubiquitin-dependent degradation via E3 ligases such as RNF5 and TRIM29—well-established suppressors of STING in mammalian systems (90, 91).

Recent cryo-electron microscopy (cryo-EM) studies have revealed that STING’s ligand-binding cleft and dimerization interface exhibit notable conformational plasticity—potential weak points where intracellular pathogens may exert pressure to disrupt signaling. These structural insights underscore the possibility that STING is not just sensed, but targeted—its molecular architecture manipulated directly to prevent immune activation (19).

The immunological destiny of a cell after STING activation is not predetermined. It hinges on the strength, duration, and context of the signal. STING can lead to interferon signaling, trigger autophagy, or initiate programmed cell death—a decision-making spectrum modulated by a finely layered regulatory logic.

In our infection model, the late-stage decline of *irf3* and *ifn*-β, despite sustained *sting1* expression, implies a checkpoint shift, potentially favoring immune escape or cellular exhaustion. This divergence points to the activation of alternative feedback circuits, such as PERK–SUMOylation axes or USP35-driven deubiquitination, which modulate STING signaling intensity and duration (92, 93).

Several intracellular pathogens have evolved to mimic these regulatory mechanisms, converging on the host’s own immune silencing strategies. *Shigella flexneri*, for instance, employs type III secretion system (T3SS) effectors that hijack the ubiquitin-proteasome pathway, leading to targeted degradation of IRF3 and indirect suppression of STING (94). This bacterial mimicry parallels the TRIM29-dependent immune dampening pathways observed in chronic infection and cancer, underscoring a shared evolutionary blueprint for immune evasion (91).

In *S. salar*, the STING1 transcript exhibited a dynamic response to infection by *P. salmonis*, marked by an early-phase induction followed by suppression by day five. This temporal arc echoes immunoevasion strategies seen in chronic viral models, where the innate system is first alerted, then dampened by pathogen-driven modulation. The loss of *irf3* and *ifn*-β transcription at late stages, despite persistent *sting1* expression, reveals a decoupling between upstream recognition and downstream effector activation—a hallmark of immune subversion. This uncoupling may arise through host-intrinsic regulators such as SUMOylated PERK, which represses STING by dampening ER stress signaling (95), or through USP35, a deubiquitinase known to stabilize STING in its inactive form (93).

Parallel strategies are employed by intracellular bacteria and viruses that degrade IRF3 or block its phosphorylation to block type I interferon induction. *Shigella flexneri*, for instance, delivers a TRIM-like ubiquitin effector through its type III secretion system, enabling targeted immune silencing (94). These bacterial tools mirror TRIM29-mediated checkpoints, where STING is marked for proteasomal clearance—suggesting a convergent evolution of host and pathogen strategies surrounding the modulation of STING signaling (91).

Furthermore, the evolutionary conservation of STING’s structural motifs—including the cGAMP-binding cleft, palmitoylation sites (Cys88/91), and LIR domains essential for autophagic engagement—across both vertebrates and invertebrates underscores its centrality in innate immunity (9, 10, 96). Strikingly, homologous STING pathways have been characterized in marine invertebrates such as shrimp, amphioxus, and ascidians, where nucleic acid sensing activates interferon-like antiviral programs, despite the absence of canonical IFN genes (90, 97–99). In these basal organisms, STING signaling is mediated by Mab21-domain proteins and NF-κB pathways, suggesting that STING’s core logic—danger sensing via cytosolic DNA—predates vertebrate interferon systems (100).

These findings decisively validate EMIR’s premise: that the STING axis is not a vertebrate invention, but a primordial molecular architecture encoded early in bilaterian evolution to confront endogenous and exogenous genomic threats. The fact that cGAMP-triggered responses have been identified in shrimp and amphioxus, triggering antiviral defenses through non-interferon pathways, confirms STING’s versatility as an immune interpreter (98, 101). In *Drosophila*, STING participates in antimicrobial defense, lipid metabolism, and mitophagy, suggesting its integration into core homeostatic systems beyond classical immunity (9, 96). The modularity of the C-terminal tail, conserved across invertebrates and mammals, allows combinatorial adaptation—with certain motifs specializing in IFN activation, and others in autophagic or NF-κB signaling (99).

Together, these invertebrate insights anchor STING as a deep-time evolutionary sentinel, validating the first layer of EMIR: the Signaling Kingdom. But evolution did not stop at cytosolic sensing. In vertebrates, STING became a regulatory hub, weaving it signaling across survival, autophagy, and death. This convergence is encoded in EMIR’s Modulatory and Proteolytic Kingdoms. Phosphorylation at Ser366/Ser358 by TBK1 enables IRF3 docking and controlled IFN transcription, while K27/K29-linked ubiquitination by TRIM10 amplifies STING’s perinuclear trafficking (102, 103). In contrast, TRIM29 or RNF5 catalyze K48-linked ubiquitination, routing STING toward proteasomal degradation and restraining overactivation (92, 104). Viruses and intracellular bacteria—including *P. salmonis*—appear to exploit these post-translational axes. Our data support a model where pathogen-triggered SUMOylation of PERK forms a rheostat, stabilizing ER stress while suppressing STING-driven death (95, 105). These mechanisms reflect evolutionary borrowing, as pathogens mimic host repressors to override STING signaling at critical inflection points (8).

At a deeper level, the Genomic Silence Kingdom orchestrates long-term repression of STING through DNA methylation and non-coding RNA-mediated mechanisms. The TMEM173 promoter is known to be hypermethylated in various cancers and chronic infections, leading to transcriptional silencing of STING unless reversed by stress-induced demethylation (106, 107). In salmonids, our transcriptomic data reveal downregulation of *sting1* during persistent infection, reflecting a similar regulatory pattern. MicroRNAs such as miR-24, IFI207, and miR-576-3p—previously implicated in STING repression in brucellosis and autoimmune diseases—are likely involved in post-transcriptional shutdown during *P. salmonis* persistence (32, 108, 109). Furthermore, long non-coding RNAs have been shown to destabilize STING mRNA, inhibit translation, or block chromatin accessibility in mammals and invertebrates (110). However, the existence and functional role of circular RNAs targeting STING, such as a putative ‘circSTING’, remain to be experimentally validated.

In *Drosophila*, epigenetic regulation of innate sensors plays an equally vital role: STING is modulated through chromatin accessibility at loci involved in mitochondrial function and immunity, reinforcing the concept that immune restraint is not a vertebrate refinement, but an ancient necessity (101).

All six EMIR Kingdoms—Signaling, Autophagic, Cell Death, Modulatory, Proteolytic, and Genomic Silence—constitute a recursive regulatory system that governs immunity not as a binary switch, but as a dynamic and symphonic network. When one kingdom is perturbed, others may compensate—or collapse—demonstrating that EMIR reflects not redundancy, but evolutionary resilience.

**Figure 12:**
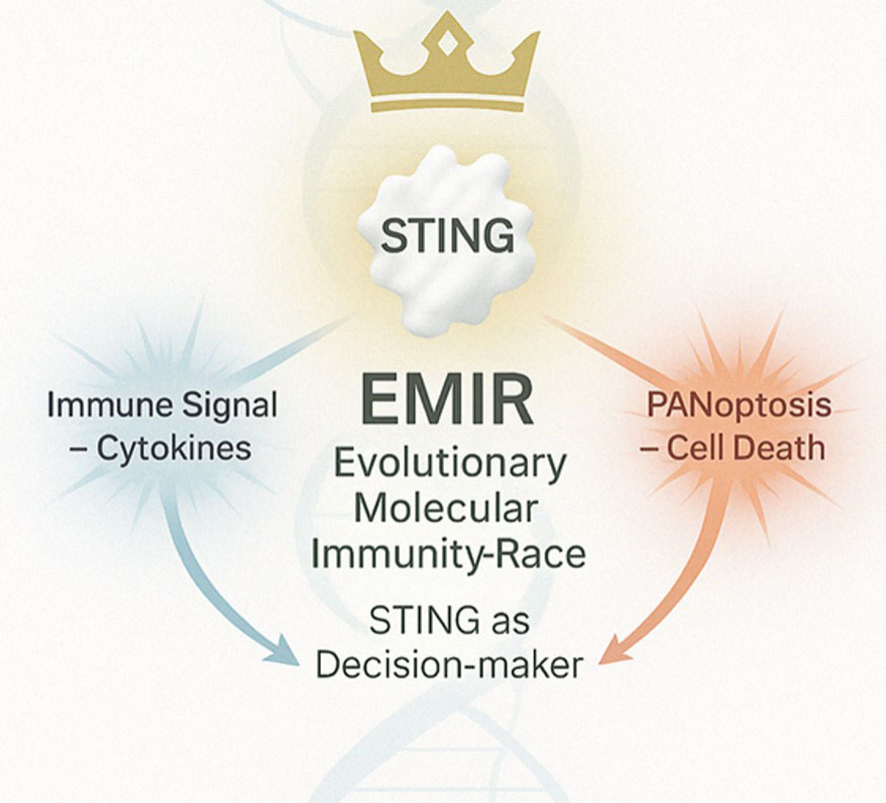
The EMIR model conceptualizes STING as an evolutionary immune decision node governed by context and coevolution. Upon activation by cytosolic DNA or cyclic dinucleotides, STING translocates from the endoplasmic reticulum to the Golgi, where it recruits and activates TBK1, which in turn phosphorylates IRF3 to drive type I interferon production and cytokine release. Simultaneously, STING may engage regulated cell death pathways—including apoptosis, necroptosis, pyroptosis, and PANoptosis—when immune equilibrium fails. EMIR (Evolutionary Molecular Immunity Race) redefines STING as a dynamic integrator of immune signaling and cellular fate, shaped by 400 million years of host–pathogen arms races.

As illustrated in **Figure 12**, the EMIR model posits that STING functions as a central decision-maker, integrating evolutionary and immunological cues to direct either cytokine production or PANoptotic cell death, depending on the selective context.

Whether *P. salmonis*, tumors, or autoimmune systems attempt to suppress STING, they target one or more of these Kingdoms. Thus, therapeutic interventions—from STING agonists to SUMOylation inhibitors or epigenetic reactivators—must be designed with kingdom-specific logic. EMIR is the key. DNA methyltransferases, such as DNMT3A, silence STING in cancer and chronic infections, a feature reversed only under acute stress or demethylation therapies (Jiang et al., 2022). At the RNA level, miR-24, IFI207, and circRNAs interact with STING mRNA or its modulators to prevent translation or degrade the message entirely (32, 108, 110). These microcircuits serve as immune “dampeners,” ensuring STING remains quiet unless an existential threat justifies its reactivation.

This deeply layered regulation echoes evolutionary necessity. In Drosophila and shrimp, similar silencing mechanisms exist, often involving cGAS-independent pathways or ancient STING homologs that modulate NF-κB, autophagy, and lipid metabolism (98, 101). In marine invertebrates, STING initiates IFN-like responses without canonical interferons—signaling through Mab21-related modules that may represent ancestral immunological logics (97, 99).

Thus, the EMIR architecture emerges not from human design but from biological continuity. It is not a metaphor, but rather a literal, stratified system of immunity, validated across phyla. STING—this single protein—recapitulates an evolutionary arms race: activated, modulated, degraded, silenced, and reborn in cycles of survival.

Our work in *S. salar* expands this framework into a translational lens. In aquaculture, *P. salmonis* manipulates multiple EMIR layers: repressing autophagy, degrading STING, silencing TMEM173—strategies that mirror cancer immune escape in humans. The solution is not to overactivate STING, but to understand which Kingdom is being subverted and restore only that layer.

In cancer, diABZI analogs, demethylating agents, or IRF3 activators can selectively reactivate silenced circuits. In autoimmune disease, palmitoylation inhibitors like H-151 or TRIM modulators help tone down runaway interferon storms. In salmonids, next-generation vaccines could include STING agonists as mucosal adjuvants, enhancing protection while avoiding overstimulation. And beyond vertebrates, in pest control, bioinsecticides could trigger PANoptotic cell death via STING, exploiting the ancestral pathways it still commands.

The EMIR framework redefines the STING pathway not merely as a receptor-mediated signaling cascade but as an integrated evolutionary system of intracellular decision-making. Through its six molecular kingdoms—Signaling, Autophagic, Cell Death, Modulatory, Proteolytic, and Genomic Silence—STING embodies a recursive architecture of immunity, shaped by deep-time coevolution with viruses, intracellular bacteria, and transposable genetic elements. This multi-tiered immune design is not an abstract construction, but a biologically validated scaffold conserved across phyla, from marine invertebrates to mammals (98, 99, 101). In *S. salar*, as in *D. melanogaster* or shrimp, the STING homolog preserves a structurally constrained CDN-binding cleft, modular activation domains, and regulatory sites for ubiquitination and SUMOylation (9, 96, 97). These molecular motifs enable the same functional versatility: the ability to sense cytosolic DNA or cyclic dinucleotides and to transduce this recognition into either immune activation, controlled self-degradation, or strategic silence (90).

This evolutionary plasticity is not a byproduct of complexity but the product of selection for contextual immunological intelligence. In our experimental model of *P. salmonis* infection, STING1 expression showed early activation and subsequent repression, mirroring suppression patterns observed in chronic viral models and tumor microenvironments (86, 89). Transcriptomic data revealed decoupling between sting1 transcription and downstream *ifn-β* and *irf3* activation at late stages, consistent with regulatory interference via PERK–SUMOylation and TRIM29-dependent proteolysis (93, 95).

This is further evidenced by the ancient conservation of autophagic-STING coupling. Non-canonical autophagy via STX17–SNAP29–VAMP8 complexes, mediated by STING’s LC3-interacting regions, has been identified in both vertebrates and invertebrates (22, 111). In shrimp and amphioxus, cGAMP-dependent activation of STING initiates antimicrobial autophagy, while *Drosophila* homologs integrate this pathway with lipid metabolism and mitochondrial homeostasis (9, 10). The presence of these mechanisms in basal lineages affirms that STING’s immunometabolic roles were foundational, and not derived adaptations limited to vertebrates.

Equally important is STING’s role in programmed cell death. Its capacity to initiate apoptosis, necroptosis, pyroptosis, or their convergence in PANoptosis, reflects its function as a terminal integrator of irreversibility (27, 28). The presence of IRF3–BAX–AIFM1 interactions and PERK–CHOP activation, even in the absence of IFN signaling, demonstrates STING’s utility when classical cytokine responses are insufficient or bypassed (112–114).

The EMIR architecture also provides a unifying model to explain therapeutic failure and success. In cancers, the Genomic Silence Kingdom is often enforced via hypermethylation of the TMEM173 promoter and suppression by microRNAs such as miR-24 (32, 107). In autoimmunity, insufficient control in the Modulatory or Proteolytic Kingdoms leads to chronic inflammation, as observed in interferonopathies like STING-associated vasculopathy with onset in infancy (SAVI) (115, 116). Interventions that selectively target these axes—whether by demethylating agents, STING agonists such as diABZI, or palmitoylation inhibitors like H-151—work not by globally activating or suppressing immunity, but by realigning specific molecular kingdoms with the cellular context (20, 89, 117).

In aquaculture, the translational implications of EMIR are no less profound. *P. salmonis* appears to manipulate multiple STING regulatory layers: suppressing autophagic flow, interfering with cytokine output, and repressing transcription of sting1 itself (25, 26). Recognizing these interventions as attacks on distinct EMIR kingdoms allows for a more precise immune restoration strategy. STING agonists can bypass upstream blockage, while epigenetic modulators may reawaken silenced loci. In this sense, EMIR offers not just a theoretical model but a practical guide—mapping pathogen interference onto targetable immune layers. Building on the evolutionary depth and regulatory elegance of STING, EMIR culminates as a framework of immune memory architecture—an operational map of how immunity calibrates its response over time, space, and stress gradients. In doing so, it reconciles ancient molecular logic with contemporary translational possibilities. This is particularly critical in hosts like *Salmo salar*, where persistent environmental pathogen exposure necessitates a flexible yet durable immune strategy.

Moreover, EMIR bridges evolutionary immunology and applied biotechnology. In fish immunogenetics, EMIR suggests selection strategies focused not only on antigen receptor diversity but also on the regulatory robustness of STING’s six kingdoms. Individuals or lineages with epigenetically resistant TMEM173 loci, robust LC3–STING–autophagy interfaces, or hyper-responsive TBK1/IRF3 modules may serve as genetic reservoirs for breeding disease-resilient stocks—propelling aquaculture into a new era of precision immuno-genomics.

In biomedical contexts, EMIR provides a lens through which to design combination therapies. For instance, STING agonists can be co-administered with inhibitors of TRIM29 or USP35 to overcome suppressive barriers in tumors. Alternatively, engineered nanoparticles carrying demethylating agents and cGAMP analogs may sequentially unlock the Genomic Silence Kingdom and then re-activate the Signaling cascade—producing durable anti-tumor immunity without autoimmunity. In vaccine science, mucosal formulations containing STING agonists can be used as adjuvants not only for viral targets but for intracellular bacteria, acting synergistically with TLRs and other PRRs to enhance innate training and memory formation.

EMIR also opens new vistas in evolutionary theory. It exemplifies how immune systems do not merely adapt but self-organize across phylogeny. Each molecular module within STING’s regulatory kingdoms reflects not only coevolution with pathogens but also integration with metabolism, stress responses, and epigenetic inheritance. It is here that EMIR transitions from being an immunological framework to an evolutionary paradigm—suggesting that natural selection acts not just on defense capacity but on regulatory plasticity and resilience.

In conclusion, EMIR transforms STING from a molecule into a metaphor for immunity itself: recursive, multi-layered, and adaptive across lineages. It proposes that the future of immunology lies not only in identifying new receptors or ligands but in decoding the layered harmonics of immune regulation. This study has not invented EMIR—it has revealed its presence, written into the architecture of STING across 500 million years of life. Through it, a new frontier in immunological logic emerges, one as elegant as it is ancient.

## 5 Conclusion

The EMIR framework repositions STING as an evolutionarily tuned molecular integrator—no longer confined to its canonical role as a cytosolic DNA sensor but elevated to the status of a recursive decision-making node capable of coordinating immune activation, metabolic restraint, and cellular self-sacrifice. Across its six regulatory Kingdoms—Signaling, Autophagy, Cell Death, Modulatory, Proteolytic, and Genomic Silence—STING operates as a dynamic system of immune intelligence, sculpted by selective pressures across 500 million years of host–pathogen interaction. This architecture allows EMIR to transcend the dichotomy between activation and inhibition, offering a unified model that explains how organisms fine-tune immunity in response to intracellular threats, damage cues, or persistent stress. The implications of EMIR are profound: it lays the groundwork for designing next-generation vaccines in which STING agonists act as precision adjuvants to enhance mucosal and systemic immunity; it informs novel antimicrobial strategies by identifying molecular checkpoints bypassed by persistent pathogens; and it offers a roadmap for cancer therapy through reactivation of epigenetically silenced STING circuits or modulation of PANoptotic cell death. Moreover, the EMIR logic can be extended to the development of bio insecticides that exploit programmed cell death pathways in pest species, or to immunogenetic selection in aquaculture, where STING signaling contributes to disease resistance in fish. In autoimmune diseases, EMIR helps clarify how the failure to restrain specific STING layers—such as palmitoylation, SUMOylation, or ncRNA-based repression—may result in interferonopathies, suggesting targeted therapies based on precise molecular inhibition. Altogether, EMIR transforms innate immunity from a reactive barrier into a strategic memory system—one capable of remembering, recalibrating, and evolving. By unveiling EMIR, this study does not propose a model: it reveals an architecture that was always present, hidden in plain sight across phyla. Through STING, nature encoded a recursive algorithm for survival, and it is through EMIR that we have begun to read its logic.

## Data Availability

The datasets presented in this study can be found in online repositories. The names of the repository/repositories and accession number(s) can be found in the article/**Supplementary Material**.
