# Supplementary material for: In evolution’s unending race: ancestral STING sensors in Salmo salar mediate intracellular bacterial detection and programmed cell death through evolutionarily conserved pathways
Source: Front Immunol. 2025 Jun 18;16:1570871. doi: 10.3389/fimmu.2025.1570871 (PMC12213456; doi:10.3389/fimmu.2025.1570871)
Supplement: Supplementary file 1 [file Table1.docx]

Supplementary Material

## Supplementary Tables

**Supplementary Table 1. List of sting1 mRNA sequences and corresponding GenBank accession numbers used for phylogenetic and comparative analyses.** This table includes the mRNA accession numbers, organism names, and associated genomic accession numbers retrieved from the NCBI database. Sequences were selected to ensure a broad representation across major vertebrate groups (fish, amphibians, reptiles, birds, and mammals). These sequences served as the basis for evolutionary and structural analyses conducted in this study.

| **Accession mRNA** | **Organism** | **Accession** |
| --- | --- | --- |
| XM_014213010.2 | *Salmo salar* | NC_059450.1 |
| XM_029771677.1 | *Salmo trutta* | NC_042969.1 |
| XM_023992127.1 | *Salvelinus alpinus* | NC_036848.1 |
| XM_039011862.1 | *Salvelinus namaycush* | NC_052323.1 |
| XM_029651675.1 | *Oncorhynchus nerka* | NW_021803039.1 |
| XM_021583458.2 | *Oncorhynchus mykiss* | NC_050571.1 |
| XM_055888463.1 | *Salvelinus fontinalis* | NC_074693.1 |
| XM_020471951.2 | *Oncorhynchus kisutch* | NC_034188.2 |
| XM_046324563.1 | *Oncorhynchus gorbuscha* | NC_060195.1 |
| XM_024428819.2 | *Oncorhynchus tshawytscha* | NC_056436.1 |
| XM_041872125.2 | *Coregonus clupeaformis* | NC_059193.1 |
| XM_023989696.1 | *Salvelinus alpinus* | NC_036846.1 |
| XM_035758961.2 | *Oncorhynchus keta* | NC_068450.1 |
| XM_047037006.1 | *Hypomesus transpacificus* | NC_061060.1 |
| XM_018669574.2 | *Lates calcarifer* | NC_066840.1 |
| XM_051419562.1 | *Dicentrarchus labrax* | NW_026136713.1 |
| XM_039813926.1 | *Perca fluviatilis* | NC_053121.1 |
| NM_001278837.1 | *Danio rerio* | NC_007125.7 |
| MN442955.1 | *Pelodiscus sinensis* | -- ^a^ |
| XM_034779649.1 | *Trachemys scripta* | NC_048305.1 |
| XM_042850749.1 | *Chrysemys picta* | NC_024225.2 |
| XM_054036523.1 | *Malaclemys terrapin* | NC_071512.1 |
| XM_041588051.1 | *Xenopus laevis* | NC_054376.1 |
| XM_040442533.1 | *Bufo bufo* | NC_053389.1 |
| XM_053718542.1 | *Bombina bombina* | NC_069504.1 |
| XM_018570186.1 | *Nanorana parkeri* | NW_017307741.1 |
| NM_001112974.1 | *Xenopus tropicalis* | NC_030679.2 |
| XM_054842303.1 | *Grus americana* | NC_072865.1 |
| XM_054288219.1 | *Melozone crissalis* | NW_026535839.1 |
| XM_032197245.1 | *Aythya fuligula* | NC_045572.1 |
| XM_059483749.1 | *Ammospiza nelsoni* | NC_080648.1 |
| MW389662.1 | *Gallus gallus* | NC_052544.1 |
| MF622062.1 | *Homo sapiens* | NC_000005.10 |
| NM_001109122.1 | *Rattus norvegicus* | NC_086036.1 |
| XM_017317994.3 | *Mus musculus* | NC_000084.7 |
| XM_027856592.1 | *Vombatus ursinus* | NW_020951350.1 |
| KT013268.1 | *Capra hircus breed Boer* | NC_030814.1 |
| NM_001046357.2 | *Bos taurus* | NC_037334.1 |
| NM_001319778.1 | *Camelus bactrianus* | NW_011517961.1 |
| XM_010718793.3 | *Meleagris gallopavo* | NC_015025.2 |

^a^ *P. sinensis* genome was not incorporated because reference genome, in assembly PelSin_1.0 as only one chromosome assembled and unplaced scaffolds.
